# Dirac magnons in a thin elemental itinerant ferromagnet

**DOI:** 10.1126/sciadv.aed9835

**Published:** 2026-07-15

**Authors:** Khalil Zakeri, Christopher Hins, Robin R. Neumann, Alexander Mook, Arthur Ernst

**Affiliations:** ^1^ Heisenberg Spin-dynamics Group, Physikalisches Institut, Karlsruhe Institute of Technology, Wolfgang-Gaede-Str. 1, D-76131 Karlsruhe, Germany.; ^2^ Institut für Physik, Johannes Gutenberg-Universität Mainz, D-55128 Mainz, Germany.; ^3^ Institut für Physik, Martin-Luther-Universität Halle-Wittenberg, D-06099 Halle (Saale), Germany.; ^4^ Institute of Solid State Theory, University of Münster, 48149 Münster, Germany.; ^5^ Institute for Theoretical Physics, Johannes Kepler University, Altenberger Strasse 69, A-4040 Linz, Austria.; ^6^ Max-Planck-Institut für Mikrostrukturphysik, Weinberg 2, D-06120 Halle (Saale), Germany.; ^7^ Donostia International Physics Center, Donostia-San Sebastian 20018, Gipuzkoa, Spain.

## Abstract

A distinct difference between graphene—an atomic layer of carbon—and conventional semiconductors is that its electrons behave as massless Dirac fermions, giving rise to unprecedented physical properties. Magnetically ordered solids host magnons, quasiparticles associated with magnetic degrees of freedom. While Dirac magnons have recently been predicted in specific insulating or rare-earth magnets, their existence in thin 3d magnets remains elusive because of the complex nature of itinerant magnetism and dimensionality effects. Here, we demonstrate the presence of Dirac magnons in a thin itinerant elemental ferromagnet. By investigating atomically designed hexagonal close-packed cobalt films, we establish that magnons in such structures resemble the Dirac electrons in graphene. We explain the physical nature of these Dirac magnons and discuss the consequences of symmetry, dimensionality, magnetic interactions, the number of atomic layers, and cobalt’s itinerant magnetism on the properties of the Dirac points. Our results pave the way for finding and engineering Dirac magnons in a variety of low-dimensional layered 3d ferromagnets and metamaterials.

## Teaser

Discovery of Dirac magnons in thin cobalt films reveals magnetic quasiparticles mimicking massless Dirac electrons in graphene.

## INTRODUCTION

The year 2025 was named the international year of quantum science and technology to celebrate the centennial of the initial development of quantum mechanics. One of the seminal contributions to quantum science was made by Paul Adrien Maurice Dirac, laying the foundation for quantum electrodynamics. In 1928, Dirac formulated an equation, which fully accounts for the special relativity in the context of quantum mechanics, describing all spin-half fermions, called “Dirac particles” (*1*). Several decades later, the Dirac electrons were found in condensed-matter systems, where the hopping between sublattices creates a coupling between the two components of the electronic wave function, mimicking the structure of the Dirac equation. The most prominent example of a material hosting Dirac electrons is graphene, a single atomic layer of carbon atoms ordered in a honeycomb lattice (*2*, *3*). The presence of Dirac electrons in several other materials, e.g., topological insulators (*4*, *5*), Dirac semimetals (*6*) and some d-wave superconductors (*7*), has now been well established (*8*).

Beyond fermions, a solid exhibits different types of bosons as the representative quasiparticles of collective excitations associated with different degrees of freedom. Examples are phonons as the representative bosonic quasiparticles of lattice vibrations and magnons as the representative bosonic excitations associated with the spin degree of freedom in magnetically ordered materials. As made possible by the coupling of different sublattices, these bosonic quasiparticles can, in principle, exhibit properties in analogy to Dirac electrons, for instance, a linear dispersion relation. It turned out that under some circumstances, one can realize Dirac bosons in specific types of materials, belonging to certain spin space groups (*9*–*13*).

Among the bosonic quasiparticles, magnons are of particular interest because they can be used to transmit information in magnonic devices (*14*, *15*) and are important for applications in quantum information technology (*16*). The experimental discovery of Dirac magnons would open up unprecedented possibilities for taking advantage of the unconventional nature of these quasiparticles for such applications. Dirac magnons with a nonzero Berry phase as well as topological magnons with a nonzero Chern number have been proposed to exist in either bulk complex magnetic materials having certain symmetries (*17*–*28*) or rare-earth magnets with hexagonal close-packed (hcp) structure such as gadolinium (Gd) (*29*, *30*). However, practical applications in the field of magnonics require high-quality thin films that can be integrated into current device technologies, such as complementary metal-oxide-semiconductor architectures. In most bulk, multielement compounds that host Dirac magnons, the structural complexity of the crystal lattice largely hinders the fabrication of nanoscale devices for terahertz magnonics (*14*). Such complex materials are often incompatible with conventional lithographic nanofabrication methods. Consequently, the discovery of Dirac magnons in thin films of elemental 3d magnetic metals would advance the fields of topological and Dirac magnonics. Furthermore, thin-film architectures potentially offer a more controllable environment for tuning the magnetic properties, which might provide greater versatility for the fundamental investigation and manipulation of these phenomena, for instance, through the exploitation of dimensionality and interface-driven effects.

In contrast to fermionic Dirac systems, where topological effects are largely governed by states near the Fermi level, bosonic Dirac points influence the dynamical response through excited states. A Dirac crossing in the terahertz regime serves as a topological singularity characterized by a quantized Berry flux, and the associated Berry curvature enters the high-frequency equations of motion of the bosonic quasiparticle wave packet (*31*). These Dirac nodes can therefore dictate the transverse trajectory and anomalous velocity of magnons when driven out of equilibrium by processes such as inelastic electron scattering, temperature gradients, hot-electron relaxation, spin injection, or other ultrafast excitation schemes. Because 3d ferromagnets, such as cobalt (Co), typically have a large exchange stiffness, with magnon energies reaching several hundred milli–electron volts (frequencies of several tens of terahertz), identifying these features in 3d magnetic thin films would likely shift the focus toward high-energy, terahertz-regime dynamics, potentially offering an accessible platform for exploring nonequilibrium topological phenomena on ultrafast (femtosecond) timescales.

The theoretical framework for magnonic band formation and the subsequent realization of Dirac-like magnonic band crossing in layered structures is fundamentally different from that governing bulk materials (*15*). This fundamental difference is rooted in the reduced dimensionality and broken symmetry inherent to the slab structure. Crucially, the appearance of multiple magnon bands in layered architectures is exclusively a consequence of the broken translation symmetry perpendicular to the plane, rather than being driven by the lattice symmetry and the presence of distinct sublattices, which are the primary mechanisms in bulk magnetic systems (*15*, *32*). Beyond the broken translation symmetry, another critical consideration is that in a thin itinerant magnetic film, the surface (or interface) layers have distinct values for the Heisenberg exchange coupling constants and the atomic magnetic moments compared to both the center and the bulk counterpart. Such layer-dependent magnetic properties arise primarily from the extension of the electronic wave function (and the associated spin density) into the surrounding vacuum or nonmagnetic substrate. This fact can impose additional symmetry constraints through changes in boundary conditions, fundamentally affecting the magnonic band protections. Together, these factors indicate that Dirac magnons in thin films are controlled by the slab symmetries rather than inherited directly from the bulk.

In most insulating multielement compounds, magnetism is typically localized, with magnetic moments confined to specific atomic sites and exchange mediated by nonmagnetic ligands. Rare-earth magnets can also be effectively treated within a localized moment framework. However, because the coupling between 4f states is mediated by itinerant electrons, the resulting interaction can be long-range in nature (*33*, *34*). In contrast, magnetism in 3d ferromagnetic metals such as Co stems from a purely itinerant band structure. In this case, the same 3d electrons are responsible for both the formation of the magnetic moment and exchange coupling. Because of the intricate landscape of these moments and interatomic exchange interactions, as well as the potential coupling between magnons and single-particle Stoner excitations, the existence of unconventional magnon manifolds in 3d magnets is not guaranteed a priori. For instance, an inspection of the calculated magnon spectra for bulk hcp Co in (*35*) suggests that such interactions may lift the magnonic Dirac degeneracy. Conversely, results obtained within a similar theoretical framework in (*36*) exhibit a persistence of these Dirac points, highlighting the sensitivity of such topological features to the specific treatment of itinerant electron dynamics. Consequently, given the itinerant nature of magnetism, the formation of Dirac magnons in 3d ferromagnetic films is not a foregone conclusion and requires experimental verification.

Here, we provide direct experimental evidence of Dirac magnons in elemental Co thin films ordered in the hcp structure. Using spin-polarized high-resolution electron spectroscopy experiments combined with the results of first-principles calculations, we show that in thin Co films of several atomic layers, one observes clear signatures of Dirac magnons, which can be efficiently excited by spin-polarized electrons. We demonstrate that despite the fact that the magnons in a thin Co film, as an itinerant magnet, are damped, they form Dirac-like band crossings and exhibit a Berry phase of $|\phi_{B}|=\pi$, as expected for Dirac magnons. We unravel the impact of both the symmetry and dimensionality on the appearance of such magnon modes exhibiting a nonzero Berry phase. It is shown that the magnonic band structure of hcp Co films with an even number of layers is composed of pairs of bands, which are the direct consequence of the peculiar symmetries of the hcp-type stacking and the finite number of layers within the structure. These pairs of bands degenerate at the high-symmetry $\bar{K}$-point of the surface Brillouin zone (BZ), forming Dirac cones. In stark contrast to the single Dirac point, whose existence is dictated by the three-dimensional bulk crystal symmetries, the observed Dirac band crossings require symmetry considerations of the whole structure, not just the symmetry of the surface. We anticipate that our results will pave the way for finding Dirac magnons in a vast variety of thin 3d ferromagnets grown on nonmagnetic substrates with hcp-type stackings. The Dirac point in bulk hcp Co could potentially be affected by interactions with the Stoner continuum. However, the thin-film geometry and high surface sensitivity of our experimental approach facilitate the detection of these features. As pointed out in (*29*), continuum scattering is expected to substantially obscure Dirac magnons in bulk Co during neutron scattering experiments. Our results demonstrate that electron spectroscopy resolves these excitations with high precision, providing a distinct advantage over bulk-sensitive probes for identifying topological magnonic features in itinerant systems. Consequently, we establish high-resolution electron scattering as an effective tool for probing and identifying the magnonic states of a thin film exhibiting a nonzero Berry phase.

Thin-film architectures provide a versatile platform for engineering magnon properties, offering a route toward potential applications in spintronics and magnonics. In principle, the linear band crossings of Dirac magnons emulate the Dirac spectrum of relativistic fermions. Consequently, they can play a central role in enabling high-fidelity spin transport, as their linear dispersion yields high group velocities and reduced scattering rates. While analogous linear magnon modes can also arise in antiferromagnets at lower energies, their contribution to Berry curvature–driven effects is typically absent once a gap opens at the linear touching point so that topological transport phenomena are not generically supported in that case. This unique combination of relativistic-like dynamics, symmetry protection, and bosonic character establishes them not only as a potential building block for energy-efficient spintronic and quantum technologies but also as a powerful probe for investigating fundamental condensed-matter physics.

It is worth pointing out that in a recent purely theoretical study, some of us have shown that magnonic Dirac crossings can emerge in engineered fcc(111) layers resulting from dimensionality and specific stacking sequences in ultrathin (two to four layers) ferromagnets (*37*). Such features are absent in the parent fcc (face-centered cubic) bulk, where the single-band magnon dispersion is continuous along all directions. As we shall show here, the Dirac nodes in hcp Co thin films represent, by contrast, discretized points of an intrinsic Dirac nodal line protected by the nonsymmorphic symmetry of the hcp lattice. This fundamental distinction establishes hcp Co as a more robust and scalable platform, as its topological properties are inherent to the crystal symmetry rather than being contingent on a specific layer-by-layer growth sequence.

## RESULTS

### Concept of Dirac magnons in hcp Co

**Fig. 1.**
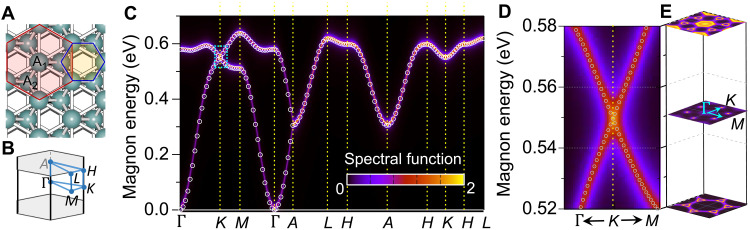
Lattice structure and the prediction of the Dirac magnons in hcp Co. (**A**) hcp lattice of Co, which includes two atoms in the conventional unit cell, marked as A_1_ and A_2_. The red and blue hexagons show the two nearly identical types of representations of the atomic structure in the vicinity of the (0001) surface, highlighting the role of different sublattices in the symmetry of this surface. (**B**) BZ of Co. The letters indicate the high-symmetry points. (**C**) Magnonic band structure of Co, calculated on the basis of first principles. The contour map represents the magnonic Bloch spectral function, and the open circles represent the magnon energies. (**D**) Magnonic band structure near the high-symmetry *K*-point, indicated by the blue dotted rectangle in (C), exhibiting a linear dispersion. (**E**) Constant energy slices at the energies in the vicinity of the Dirac point.

In the bulk crystal of Co, the atoms are ordered in an hcp lattice. In the conventional unit cell basis, the structure is characterized by two hexagonal lattices, which are shifted with respect to each other by the displacement vector ($\frac{2}{3}$, $\frac{1}{3}$, $\frac{1}{2}$) (see also text S1). Considering the symmetries of the hcp lattice, one recognizes that in the conventional notation, it is necessary to include two atoms to describe the structure. These atoms are marked by A_1_ and A_2_ in Fig. 1A, where a schematic illustration of the hcp lattice of Co is presented. The corresponding BZ is shown in Fig. 1B. Another consequence of the symmetry operators of this lattice is that the magnonic band structure of the system has to exhibit two bands. The magnonic band structure of hcp Co calculated on the first-principles basis is presented in Fig. 1C. Both the magnonic Bloch spectral function and magnon dispersion relation are shown over the entire BZ (see Materials and Methods for a detailed description of the first-principles calculations). The calculated bulk magnon dispersion for hcp Co (Fig. 1C) is in very good agreement with previous theoretical reports. Specifically, our results for the acoustic and optical branches along the high-symmetry directions closely reproduce the ab initio calculations in (*35*, *36*, *38*–*42*). This validates our computational framework, providing a reliable foundation for the thin-film calculations presented in the following sections.

The magnonic band structure exhibits two bands, well separated at the zone center (Γ-point). These bands cross at the high-symmetry *K*-point. At this high-symmetry point, along the *K*-*H* direction, and within the entire *A*-*L*-*H*-*A* plane, the two bands fully degenerate. A careful inspection of the magnonic band structure indicates that in the vicinity of the crossing point (*K*-point), the magnons exhibit a linear dispersion relation, similar to the Dirac electrons in graphene. The Dirac point is located at *K* (0.55 eV). This is clearly visible in Fig. 1D, where a magnified part of the magnonic band structure near the *K*-point is shown. The presence of the Dirac magnons in this system is also demonstrated in Fig. 1E, where the constant energy cuts are presented. The presence of Dirac magnons in this system is a direct consequence of the symmetries of the hcp lattice and the presence of different magnetic sublattices (see below). Similar to other hcp ferromagnets, e.g., Gd (*29*, *30*), the magnonic bands of Co resemble the electronic bands of graphene, as the bosonic Hamiltonian of magnons in this system can be interpreted in analogy to the fermionic Hamiltonian of electrons in graphene.

The point group symmetry of the hcp lattice of Co is $D_{6h}$. It has a center of inversion, a screw symmetry, and a threefold symmetry about the *c* axis. Moreover, in the absence of spin-orbit coupling, the single-particle magnon Hamiltonian is also invariant under an effective time-reversal symmetry. The simultaneous presence of these symmetries not only leads to fully degenerate magnon bands along the *A*-*L-H*-*A* symmetry path but also to the formation of Dirac points at the high-symmetry *K*-points and nodal lines along the vertical direction to the *H*-point of the BZ. The symmetry-driven nature of the magnon Dirac band crossings can be demonstrated by calculating the Berry phase along a closed loop encircling the nodal line. The results indicate that for the contours about the *K*-points, the band has a Berry phase of $|\phi_{B}|=\pi$ (see Materials and Methods for details of the calculations).

### Magnons in hcp thin ferromagnetic films with a finite number of layers

To investigate the potential formation of Dirac magnons in hcp films with various surface orientations, we conducted a symmetry-based analysis. Our results indicate that the thin hcp(0001) films are the best candidate for realization of Dirac magnons (see text S1 and table S1).

As noted earlier, the point group symmetry of the hcp lattice of Co is $D_{6h}$. However, once a thin film is formed, the symmetry is lowered because of the presence of the surface and interface and the finite number of atomic layers (hereafter referred to as $C_{3v}$ symmetry; see also Fig. 1A). In principle, unlike bulk hcp Co, thin hcp(0001) Co films exhibit broken translational symmetry perpendicular to the surface while preserving hexagonal symmetry within the film plane. This structural environment allows for the control of sublattice coupling through epitaxial strain or layer thickness, providing a direct mechanism to tune the energy and dispersion of the Dirac crossing for any practical application. Consequently, we examined the possible formation of Dirac magnons in an epitaxial film of hcp Co(0001) with the thickness of 20 monolayers (MLs) grown on W(110).

**Fig. 2.**
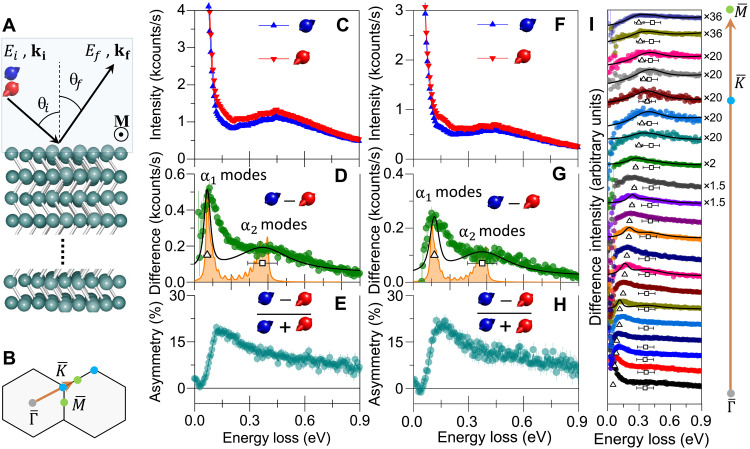
SPHREELS spectra recorded on a 20-ML thick film of hcp Co on W(110). (**A**) Scattering geometry used in the experiment. *E_i_*$E_{i}$ and *E_f_*$E_{f}$ denote the energy of the incident and scattered electron beams, respectively. $k_{i}$ ($\theta_{i}$) and $k_{f}$ ($\theta_{f}$) denote the momentum (angle) of the incident and scattered electron beams, respectively. (**B**) Propagation direction of the magnons in the reciprocal space for the spectra shown in (C) to (I). (**C** to **H**) Typical SPHREELS spectra recorded on 20-ML Co on W(110) at the incident energy of *E_i_* = 4 eV at room temperature. The spectra shown in (C) to (E) and (F) to (H) are recorded at the wave vector transfers of ∣Q∣=0.5 and 0.6 Å^−1^, respectively. The spin-polarized spectra are shown in (C) and (F), the difference spectra are shown in (D) and (G), and the asymmetry spectra are presented in (E) and (H). The spectra shown in red and blue were recorded with the spin polarization vector of the incident electron beam being parallel and antiparallel to the scattering plane’s normal vector $\hat{n}$ (or the magnetization M), respectively. In (D) and (G), the spectral function calculated on the basis of first principles is also shown by the orange color for a comparison. One recognizes the presence of two classes of magnon modes. These are denoted by $\alpha_{1}$ and $\alpha_{2}$. (**I**) Series of the difference spectra recorded along the $\bar{\Gamma }-\bar{K}$ high-symmetry direction (and beyond). The solid black curves in (D), (G), and (I) contain two peak functions mimicking the $\alpha_{1}$ and $\alpha_{2}$ modes and may be used as the guide to the eye. The square and triangular symbols represent estimated peak positions extracted from the experimental data using the approach explained in text S2. For an extended version, see fig. S2.

The details of the sample preparation and characterization are provided in Materials and Methods. The magnons were probed by means of spin-polarized high-resolution electron energy-loss spectroscopy (SPHREELS) (*43*–*46*). The scattering geometry used in the experiment is shown in Fig. 2A. In this particular example, the magnon propagation direction was along the Co(0001)[$11\bar{2}0$] direction, corresponding to the $\bar{\Gamma }-\bar{K}-\bar{M}$ direction of the reciprocal space, as indicated in Fig. 2B.

Typical SPHREELS spectra are presented in Fig. 2 (C to H). The spectra were recorded at wave vector transfers of ∣Q∣=0.5 and 0.6 Å^−1^ along the $\bar{\Gamma }-\bar{K}$ direction and using an incident electron energy of *E_i_* = 4.0 eV. The red and blue colors represent the cases in which the spin of the incident beam was parallel and antiparallel to the scattering plane’s normal vector $\hat{n}$, respectively (*44*, *45*). The broad features observed in both spin channels of the spin-polarized spectra are attributed to non–spin-flip excitations. Given the metallic character of the sample and the abundance of electrons near the Fermi level, several processes are possible, including short-lifetime single-particle excitations (*47*). The corresponding difference $I_{↓}-I_{↑}$ and asymmetry ($I_{↓}-I_{↑}$)/($I_{↓}+I_{↑}$) spectra for ∣Q∣=0.5 Å^−1^ (0.6 Å^−1^) are shown in Fig. 2D (Fig. 2G) and Fig. 2E (Fig. 2H), respectively. Both difference and asymmetry spectra include all the possible magnon modes of the system. The spectra clearly exhibit a multimode character. Specifically, they feature two intense magnon modes localized primarily in the two topmost layers, accompanied by several satellite peaks attributed to bulk modes residing mainly in the inner layers (*15*, *32*, *45*, *48*–*52*). The reduced visibility of the higher-energy modes in the asymmetry spectra can be traced directly to the mathematical definition of the asymmetry itself. Because the total intensity appears in the denominator, it is expected that these magnon peaks are inherently less pronounced in the asymmetry spectra (see text S2). To identify the origin of the magnon modes observed in the experiments, we performed first-principles calculations of the magnonic Bloch spectral function (MBSF). The MBSFs associated with the top two layers are shown in Fig. 2 (D and G) for two representative cases, corresponding to the experimental spectra. Very similar to the experiment, one clearly observes the presence of two classes of magnons, denoted as $\alpha_{1}$ and $\alpha_{2}$ modes. Note that in the adiabatic calculations presented here, the lifetime broadening due to the Landau damping has not been taken into consideration. Hence, the magnon modes appear as sharp excitations. Here, the most important result is that the presence of the two classes of magnons in the system (referred to as $\alpha_{1}$ and $\alpha_{2}$ modes) is a direct consequence of the hcp structure, as the modes are predominantly localized on one of the sublattices $A_{1}$ and $A_{2}$. To better follow the dispersion relation of these two classes of magnons, two peak functions were used, each of which mimics one of the two classes (*53*). Details are also provided in text S2 and fig. S1. The peak positions extracted from the experimental data, following the procedure detailed in text S2 and fig. S2, are represented by squares and triangles.

In Fig. 2I, a set of difference spectra, recorded at various wave vectors, is presented, indicating a clear dispersion of all the magnon modes. In addition, the peak functions representing the $\alpha_{1}$ and $\alpha_{2}$ modes are also shown as a guide to the eye. Figure 2I demonstrates that in contrast to the modes denoted by $\alpha_{1}$, those marked as $\alpha_{2}$ exhibit a rather weak dispersion relation. The two classes of modes degenerate at the high-symmetry $\bar{K}$-point of the surface BZ. The data also reveal a distinct band crossing of these modes at the $\bar{K}$-point, within the lifetime broadening, accompanied by a linear dispersion relation both above and below this point (see also fig. S2).

**Fig. 3.**
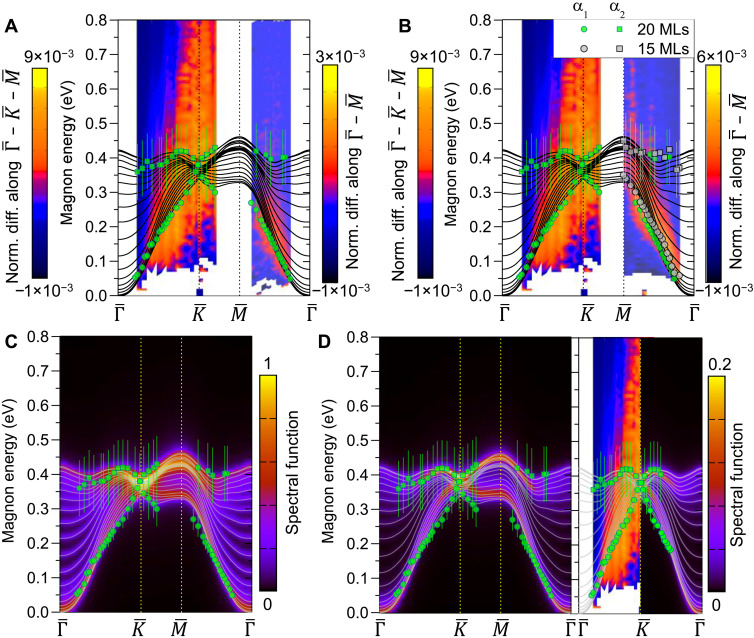
Magnon dispersion relation of thin hcp Co films on W(110), with thicknesses of 15 and 20 MLs. (**A**) Intensity map of a 20-ML Co film. The normalized difference spectra are plotted as the color map. The spectra were normalized to the elastic peak. The apparent enhancement of the high-energy background intensity near the $\bar{K}$-point is partly a consequence of this normalization procedure and partly due to the presence of single-particle excitations within the Stoner continuum. The magnon energies of the $\alpha_{1}$ and $\alpha_{2}$ modes are shown by the solid circles and squares, respectively. The error bars (standard errors) are shown by the vertical lines. The results of ab initio calculations are also shown by solid curves. (**B**) Comparison between the results of the 20-ML sample and the sample with the film thickness of 15 MLs. Note that the results of the 15-ML sample are shown along the $\bar{\Gamma }-\bar{M}$ direction only. To account for the excitation cross section, the result of Q>1.3 Å^−1^ is scaled so that the background intensity matches the results recorded at Q=1.2 Å^−1^. All spectra were recorded at room temperature. Calculated magnon BSF projected onto (**C**) all 20 layers and (**D**) the top two atomic layers. The solid circles and squares indicate the peak positions of the two main magnon modes $\alpha_{1}$ and $\alpha_{2}$, respectively. The results of the 20- and 15-ML samples are shown by the green and gray colors, respectively. For an extended version, see also fig. S3.

To verify the Dirac properties of the magnon modes of the system, we analyzed the intensity map of these magnons in the energy-momentum space. The intensity map along the main symmetry directions of the surface BZ is summarized in Fig. 3. The results are compared to those of ab initio calculations. In Fig. 3A, the results of a 20-ML sample are presented and are compared to the magnon bands calculated on the basis of first principles. The solid symbols represent the magnon energies of the two modes, and the vertical lines represent the upper limit of their error bars. As discussed in text S2, because of the presence of several magnon modes and the fact that each magnon mode has a certain lifetime broadening and amplitude, it is unrealistic to try to separate all these magnon modes. Instead, we estimate the magnon energies by analyzing the center of mass of the maxima observed in the spectra. The presence of 20 magnon bands in the calculations is a direct consequence of the confined geometry in the direction perpendicular to the layers, leading to a discretization of the magnon Dirac nodal line. To unravel the origin of the observed magnon modes, experiments were performed on a thinner sample with the thickness of 15 MLs only along the $\bar{\Gamma }-\bar{M}$ direction. Along this symmetry direction, neither a band crossing nor a nonzero Berry phase is expected. The magnons are anticipated to behave classically, as in standard slab (thin film) systems. The SPHREELS spectra recorded on this sample are presented in fig. S4. The resulting magnonic spectrum over the energy-momentum space is presented in Fig. 3B, together with the results of the 20-ML film. The similarity of the data for the two samples along this symmetry direction ensures that the probed magnon signal can be attributed to the topmost two layers, with some contributions from the layers located in the inner part of the Co film. Further details are provided in text S3 and fig. S5. This fact is also supported by the calculated layer-resolved MBSF and layer-resolved magnonic density of states (MDOS). The MBSF projected onto all 20 layers is shown in Fig. 3C. The spectral function projected onto the two topmost layers is presented in Fig. 3D, further confirming that the main contribution to the measured spectra originates from the magnons mainly localized in the two topmost surface layers. The same behavior is also observed in Fig. 2 (D and G), where the spectral function projected onto the two topmost surface layers for two representative wave vectors is shown. For an extended discussion, see text S3 and S4 and figs. S5 to S7.

It is crucial to recognize that the presence of Dirac magnons in thin magnetic films is not obvious, even when their bulk counterpart is theoretically predicted to exhibit them. A critical consideration overlooked in previous experimental and theoretical investigations is the inherent reality of layered structures: The surface (or interface) layers have distinct values for the Heisenberg exchange coupling constants and the atomic magnetic moments compared to both the bulk and inner layers of the slab. The layer dependence of magnetic parameters is well established, as confirmed by both first-principles calculations and experimental results (*45*, *48*, *54*, *55*). It is attributed to the extension of the electronic wave function, and consequently, the spin density, into the vacuum (or the nonmagnetic substrate, if present). Our calculations fully incorporate all these effects. This layer-dependent phenomenon can enhance the magnetic symmetry of the system, as the ratios of the exchange parameters and magnetic moments enter the Hamiltonian (see also text S5). As a result, the modes associated with the surface/interface do not simply appear below the bulk continuum, as one would expect from the reduced mean fields close to the surface. Instead, they appear at the edge of the bulk continuum and can therefore hybridize with the bulk modes (see also text S5).

As a consequence of the itinerant nature of magnetism in Co, the magnons are damped in this system. The main source of damping of high-energy exchange-dominated magnons in itinerant magnets is due to their decay into electron-hole pair excitations, known as Stoner pairs, as has been confirmed by the calculations accounting for this so-called Landau damping (*35*, *36*, *56*–*60*). Irrespective of their strong damping, the magnons still exhibit Dirac-like properties in the vicinity of the $\bar{K}$-point, as seen in the intensity maps presented in Fig. 3. A similar behavior can also be observed in the constant energy slices of the magnonic spectrum.

**Fig. 4.**
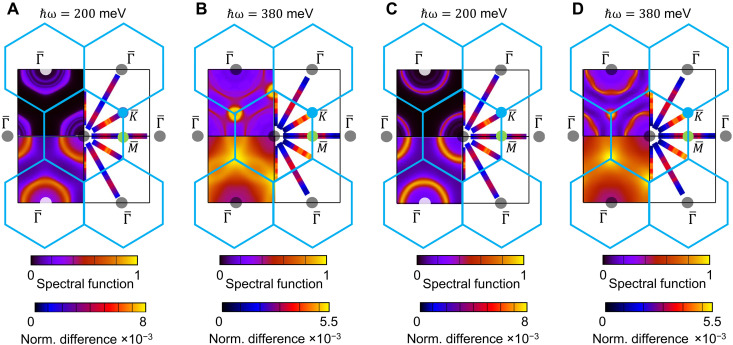
Constant energy slices away and in the close vicinity of the Dirac point. (**A**) Intensity map at the energy of *ℏ*ω = 200 meV. The experimental data (right quarters) are shown together with the calculated MBSF (upper left quarter). In the lower left quarter, the calculated spectral function is broadened by 42 meV. (**B**) Intensity map near the Dirac point energy (*ℏ*ω = 380 meV). The experimental results (right quarters) are compared with the calculated MBSF (upper left quarter). In the lower left quarter, the calculated spectral function broadened by 80 meV is shown. The projected MBSFs onto the two topmost layers are shown in (**C**) and (**D**) and are compared to the experimental magnon spectrum. The results of *ℏ*ω = 200 meV and *ℏ*ω = 380 meV are shown in (C) and (D), respectively.

In Fig. 4, we show the constant energy slices away and close to the Dirac point energy. The results for the energy of *ℏ*ω = 200 meV, away from this point, are shown in Fig. 4A. The experimental data, shown in the right quarters, are compared with the calculated MBSF, shown in the upper left quarter. A simple but practical way to include the effects of Landau damping in MBSF is to broaden the calculated MBSF by a value corresponding to the broadening observed in the experiment. Such a consideration would mimic the effect of the Landau damping on the spectral function. The results of this analysis are presented in the lower left quarter of Fig. 4A, where a broadening of 42 meV is assumed. Both experimental and computational results show the expected ring-like pattern of the intensity map centered around the zone centers.

The results for the Dirac point are presented in Fig. 4B. Both the experimental and computational results are presented for the energy *ℏ*ω = 380 meV. While the spectral function as calculated on the basis of first principles is shown in the upper left quarter, the broadened MBSF by 80 meV is shown in the lower left quarter. The results further confirm the presence of the Dirac point as a high-intensity area located at the $\bar{K}$-point. The Dirac point is clearly visible in the calculated MBSF without considering the broadening caused by the damping. The presence of the Landau damping smears out the spectra. The calculated results of MBSF exhibit a high intensity at this point even after considering the damping, similar to the experimental results. The intensity at the other parts of the surface BZ is lower than at this point. In particular, the calculations predict that the map along the $\bar{\Gamma }-\bar{M}$ direction and at the $\bar{M}$-point should exhibit a lower intensity compared to the $\bar{\Gamma }-\bar{K}$ path and the $\bar{K}$-point. This behavior is clearly observed in the experimental results. A similar conclusion can be drawn by looking at the MBSF projected onto the two topmost layers. The results of the MBSF projected onto these layers are presented in Fig. 4 (C and D), where we show the spectral function with and without considering the broadening associated with the Landau damping. The better agreement between the results of the calculations when the spectral function is projected onto the topmost layers and those of the experiment indicates, once again, that the main contribution to the experimental spectra originates from the two topmost surface layers. Because of the fact that SPHREELS is mainly a surface probe technique, such a behavior is expected.

## DISCUSSION

### Impact of itinerant electrons and the number of atomic layers

It is important to notice that because of the itinerant nature of magnetism of Co, the pattern of both the Heisenberg exchange parameters and magnetic moments across different layers is no longer uniform. It is often observed that both of these quantities are enhanced in the vicinity of the surface. This is attributed to the contribution of different orbitals to the interatomic Heisenberg exchange interaction and the spin-density redistribution near the surface (see also Materials and Methods). One of the prime consequences of this fact is that the surface magnon modes of the Co films are located in the close vicinity or within the bulk modes (instead of being located far below them), as observed in Figs. 2 and 3. An extended discussion is provided in text S5 and S6 and figs. S7 and S8.

As seen in Fig. 4 (B and D), the Dirac point is not as sharply defined as the corresponding feature presented in Fig. 1 (D and E) for bulk Co calculations, excluding damping. This finding aligns with results for the bulk itinerant magnet FeSn (*25*) and has been attributed primarily to the Landau damping of these magnon modes. The results indicate that the Stoner continuum does not cause a detectable gap in the two-dimensional (2D) Dirac crossings in thin Co films. Given that thin films can in principle exhibit a different Stoner continuum than the bulk, this is reasonable (*35*, *59*).

**Fig. 5.**
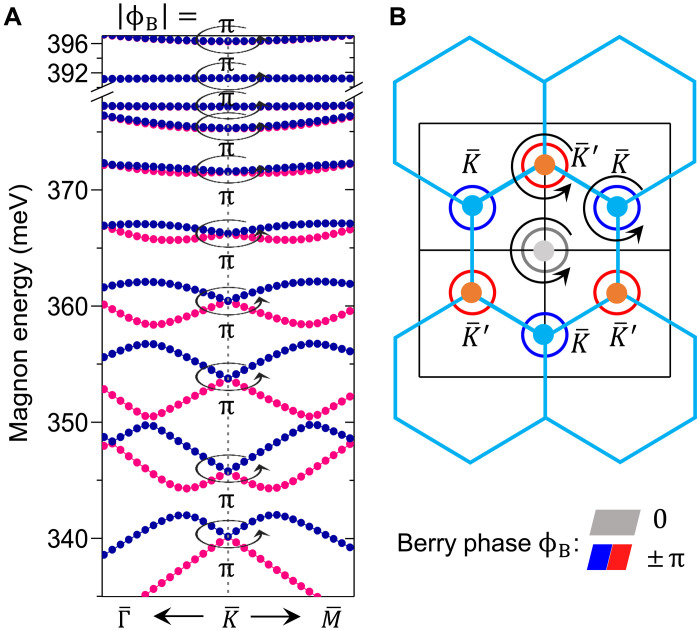
Berry phase of the magnonic bands in the vicinity of the Dirac points. (**A**) Magnonic band structure of a 20-ML Co film near the $\bar{K}$-point. The magnonic band structure comprises pairs of bands (shown in red and blue) characterized by a quantized Berry phase of $|\phi_{B}|=\pi$. At the Dirac node, these branches exhibit a degenerate crossing where the eigenstates undergo an instantaneous exchange of character. (**B**) Distribution of the Berry phase in the 2D reciprocal space. The magnon band pairs at the high-symmetry $\bar{K}$- and ${\bar{K}}^{\prime }$-points have a Berry phase of $|\phi_{B}|=\pi$. These points exhibit opposite signs of the Berry phase, indicated by the red and blue color mapping. The arrows denote the integration contours used to calculate the Berry phase around the high-symmetry points.

Beyond the effects of Landau damping, another contributing factor is the fact that layered systems composed of a finite number of layers often exhibit multiple Dirac points in the vicinity of the $\bar{K}$-point (see, e.g., Fig. 5A). A careful analysis of the magnonic band structure revealed that the appearance of these points, instead of a single Dirac point, is a result of the confinement in the direction perpendicular to the layers and the even number of layers in the 20-ML film, in which the A_1_/A_2_ stacking sequence of hcp is preserved.

In general, for a film composed of a finite number of layers, there exist several “confined magnon modes.” The energy spread of these modes is mainly determined by the strength of interlayer Heisenberg exchange interactions and is approximately given by the projection of the bulk magnon bands onto the surface. In the vicinity of the $\bar{K}$-point, the confined magnon modes are spread over an energy window of about 60 meV. This is in agreement with the weak energy dispersion of the bulk magnonic band structure along the *K*-*H* symmetry path, starting from 550 meV at the *K*-point up to about 600 meV at the *H*-point (over an energy range of about 50 meV; see Fig. 1C and also fig. S8). Owing to the fact that in a slab of 20 layers, the inversion symmetry of the bulk is approximately preserved if substrate effects can be neglected, the magnonic bands pair up and form Dirac points. These Dirac points are spread over an energy range of about 60 meV. The symmetry-driven Dirac nodal line of the hcp Co bulk is transformed into several discrete Dirac points at the $\bar{K}$-point of the surface BZ (see also text S6). Ultimately, the Berry phase in the vicinity of these crossing points confirms their Dirac nature.

The analysis of the magnetic parameters revealed that the appearance of these points, instead of a single Dirac point, is a result of the nonzero out-of-plane interatomic exchange parameter describing the interaction between the sublattices of the same kind (A_1_ and A_2_ in Fig. 1A). If the exchange parameters describing this interaction are set to zero, the magnonic band structure of the bulk system will be flat along the *K*-*H* high-symmetry line and, consequently, all the Dirac points would degenerate, forming a single Dirac point.

### Magnon Berry phase and symmetry-driven properties

As discussed above in the case of 20 MLs, the inversion symmetry of the whole stack is approximately preserved (because of the A_1_/A_2_ stacking of layers) if substrate effects can be neglected. Hence, the idealized or freestanding Co system meets the symmetry requirements for the formation of Dirac points. To ensure that these points are of Dirac nature, we calculated and analyzed the Berry phase of all the magnon bands using exchange parameters calculated from first principles for a freestanding 20-ML Co film. We use a linear spin-wave theory framework to treat the topological properties of layered structures. Unlike standard computational packages optimized for three-dimensional bulk crystals or idealized 2D model systems, our implementation seamlessly integrates complex layer-dependent interactions. It enables the precise determination of the full magnonic spectrum, including the band-resolved Berry phase and associated topological properties (see Materials and Methods). The results, presented in Fig. 5, clearly indicate that for the contours around the $\bar{K}$(${\bar{K}}^{\prime }$)-point, where the Dirac points are located, all the observed magnon modes have a Berry phase of $|\phi_{B}|=\pi$. The contours around the other symmetry points of the surface BZ, e.g., the zone center $\bar{\Gamma }$-point, result in a Berry phase of zero. As seen in Fig. 5A, the nontrivial Berry phase of $|\phi_{B}|=\pi$ around the $\bar{K}$(${\bar{K}}^{\prime }$)-point indicates that the single-particle eigenstates of the Heisenberg Hamiltonian cannot be smoothly defined without a singularity. While the magnon energy dispersion evolves continuously through the $\bar{K}$-point, the underlying eigenstates are mathematically singular at the crossing. The presence of a Dirac node necessitates an abrupt exchange in the sublattice character (or pseudospin) of the magnon mode upon traversing this crossing point. This sublattice swap manifests as an intensity winding, the consequences of which are observed in our experimental data (see below). The opposite values of the Berry phase at the $\bar{K}$ and ${\bar{K}}^{\prime }$ valleys are inherent to the bipartite lattice of the investigated system and can be traced back to its fundamental symmetries (text S1). The weak inversion-symmetry breaking perturbations expected to exist in any real sample because of the substrate can be expected to gap out the Dirac cones, rendering them massive and causing a finite Berry curvature. These gaps are, however, expected to be smaller than the broadening associated with the magnon lifetime and thus remain unresolvable.

The Berry phase of ±π is expected to result in an intensity winding of the spectra when encircling the Dirac point in bulk hcp ferromagnets (*29*, *30*). One would also expect that a similar behavior should be observed for a thin hcp slab composed of a finite number of layers (see, for example, text S7). However, such an intensity winding is supposed to be rather weak because of the thin film geometry, the nature of electron-magnon interaction, the damping effects, and the presence of single-particle excitations. The experimental data supporting this expectation are presented in fig. S9 for a 20-ML Co film. We emphasize that for the 15-ML film (or any film with an odd number of layers), a Berry phase of ±π for the surface-localized magnon bands is not guaranteed by the symmetry of the slab. However, as clearly visible from the calculated magnon bands (see, for example, fig. S7), both thicknesses exhibit several pairs of magnons that cross at the $\bar{K}$-point, forming Dirac-like crossings. These crossed magnon bands can still have a nonzero Berry phase mainly derived from the local symmetry of the system.

It is not straightforward to observe an even-odd effect in the intensity maps of these rather thick films discussed here. The presence of several Dirac-like crossings and the Landau damping in Co does not allow to resolve individual Dirac cones. Therefore, one expects the experimental intensity map to be practically similar for films with an even or odd number of layers.

At room temperature, a Dirac crossing at 380 meV is not populated under thermal equilibrium. However, within the framework of magnetism, the physical significance of a Dirac point may not be defined solely by its equilibrium occupation but rather by its role as a source of Berry curvature, which governs magnon dynamics through the semiclassical equations of motion (*31*), in the dynamical response. Consequently, the presence of such a node and the resulting anomalous magnon velocity may carry fundamental implications for the high-frequency dynamical response. While the treatment of these dynamical effects is beyond the scope of the present study, the observed nonzero Berry phase provides the necessary foundation for further investigation of such behavior.

We note that our focus here is on the integrated phase winding of the magnon eigenstates around the Dirac points. In a more general context, this is a manifestation of the Berry curvature, for which the observed ±π Berry phase is the global signature. This curvature can, in principle, govern magnon dynamics through the semiclassical equations of motion (*31*). Within this framework, the velocity of a magnon wave packet is determined not only by the conventional group velocity derived from the dispersion gradient but also by a particularly interesting contribution known as the anomalous velocity. This latter component is proportional to the Berry curvature and may become considerable under specific conditions (*31*). Consequently, while trivial magnons elsewhere in the surface BZ follow trajectories dictated solely by the dispersion gradient, magnons near the $\bar{K}$-point can, in principle, have an anomalous velocity. Such behavior could be important for systems with a mass term in the Hamiltonian caused, for example, by symmetry breaking. This could potentially provide a “topological filter” for the nonequilibrium response when magnons are excited in the vicinity of the Dirac point in a momentum-resolved excitation scheme (*61*). We note that while the treatment of these dynamical effects is beyond the scope of the present study, the observed nonzero Berry phase provides the necessary foundation for further investigation of such behavior.

We underscore that while broad spectral features are an inherent physical property of itinerant magnets, our identification of a 2D Dirac crossing is supported by a self-consistent framework of three independent experimental signatures: (i) the apparent merging of peak centers tracing the coalescence of the $\alpha_{1}$ and $\alpha_{2}$ branches across the entire surface BZ, (ii) the emergence of an intensity maximum at the $\bar{K}$-point at the Dirac node energy, and (iii) the experimental detection of the consequences of the intensity winding about the $\bar{K}$-point, confirming a nonzero Berry phase, a robust hallmark of Dirac magnons that persists despite Landau damping (see also text S8).

In the absence of spin-orbit coupling giving rise to, e.g., antisymmetric interactions [Dzyaloshinskii-Moriya interaction (DMI)], hcp films do not exhibit nontrivial magnon Chern numbers (similar to bulk hcp ferromagnets). This is because the Berry curvature integrates to zero over the 2D BZ because of symmetry constraints, as is clearly apparent from Fig. 5B. However, beyond its fundamental importance, the Dirac crossing in hcp Co thin films can serve as the essential parent phase for engineering magnonic Chern insulators with a nonzero Chern number. By using interfacial DMI in Co/heavy metal, this symmetry-protected degeneracy can be lifted to realize a topological spectral gap (*62*–*66*). This effect becomes increasingly important in thinner films, as the interfacial DMI strength scales inversely with the ferromagnetic layer thickness. Moreover, the linear dispersion of these ∼85- to 95-THz branches may provide a foundation for ultrafast signal transport, while the concentration of Berry curvature may facilitate the observation of the magnon Hall effect in the terahertz regime. Because the energy of the Dirac node is governed by interlayer exchange interactions, the system remains tunable via strain or layer thickness, establishing hcp Co films as a potential room-temperature platform for high-frequency topological magnonics.

We anticipate that Dirac magnons should be observed in other thin films of 3d magnets ordered in the hcp structure. This would open up possibilities to use such magnons for practical applications. In this regard, one obstacle is the presence of the Landau damping and, consequently, the short lifetime of these magnons in itinerant magnets. However, one may overcome this obstacle by tuning the Landau damping via engineering of the electronic structure of the system. A strategy has been proposed in (*14*, *59*, *67*, *68*) and has successfully been implemented to similar systems. Likewise, the excitation frequency of these magnons can also be tuned by alloying with other elements, tuning the interatomic distances by epitaxial strain or growing magnetic superlattices composed of alternating atomic layers made of different magnetic elements (*67*). Besides concentrating on avoiding its detrimental Landau damping effects, electron-magnon interactions could also be addressed within an effectively non-Hermitian framework and be analyzed for their non-Hermitian topology, as has been proposed in (*69*) for magnon-magnon interactions.

One of the primary advantages of 3d transition metals is their compatibility with conventional nanofabrication techniques, which facilitates the integration of Dirac magnons into the burgeoning field of terahertz magnonics. However, the use of metallic ferromagnets as a platform for topological magnonics involves a unique set of technological trade-offs. While the presence of mobile charge carriers introduces considerations such as ohmic dissipation and potential parasitic electronic signals, these may be balanced by the advantages of metallic systems. Specifically, the coexistence of charge and spin degrees of freedom allows for the direct electrical control of magnons via the spin Hall effect and related phenomena, enabling the development of integrated spintronic-magnonic architectures.

Furthermore, compared to complex magnetic insulators, elemental metallic films offer greater scalability and facilitate nanopatterning with atomic-layer precision. These features, combined with the room-temperature stability and high-energy Dirac magnons reported here, position 3d transition metals as a potential candidate for on-chip topological information processing. In a similar manner, it is expected that artificial periodic magnetic metamaterials ordered in an hcp structure will also exhibit such Dirac magnons.

Beyond transport, the discovery of Dirac magnons at such high energies (380 meV ≈ 90 THz) may be important for the fundamental understanding of ultrafast spin-lattice coupling and electron-spin dynamics. It has been experimentally demonstrated that the relaxation of hot electrons in Co films is dominated by the emission of high-energy magnons on a femtosecond timescale (*70*). These high-energy excitations provide a primary microscopic channel for ultrafast spin-flip processes (*46*, *71*). It might therefore be relevant to investigate the impact of a Dirac crossing in this energy regime to determine how it might alter the scattering phase space available for such relaxation. Because the Dirac point acts as a topological singularity in the magnon spectrum, it may, in principle, influence the rate and efficiency of spin-dependent electron thermalization. Our identification of Dirac magnons within the high-energy manifold of hcp Co films may thus provide a basis for more detailed microscopic investigations of these ultrafast processes and the possibility of their selective manipulation.

We anticipate that our results will inspire new approaches for investigating laterally resolved magnon spectra. Potential methodologies to achieve this include (i) using well-ordered self-organized magnets (*53*), (ii) using locally resolved tunneling spectroscopy (*72*), and (iii) leveraging the recently developed technique of laterally resolved electron energy-loss spectroscopy within an electron microscope (*73*).

In conclusion, using spin-polarized high-resolution electron spectroscopy experiments and first-principles calculations, we provided direct experimental and computational evidence of Dirac magnons in elemental Co thin films with an hcp lattice structure. It was demonstrated that the magnonic spectrum of thin hcp Co films of several atomic layers shows clear signatures of Dirac-like band crossings. Such nontrivial magnonic states having a nonzero Berry phase can be efficiently excited by means of spin-polarized electrons despite their notable Landau damping. Berry phase and protection properties of magnons in hcp films are governed by the presence of the $C_{3v}$ and time-reversal symmetry, which, combined with the inversion symmetry for an even number of layers, leads to the formation of Dirac magnons with a Berry phase of ±π at the high-symmetry $\bar{K}$-point of the surface BZ. In essence, the interplay of the thin-film geometry and unique electronic properties (specifically those that suppress the influence of Stoner excitations) facilitates the formation of the 2D Dirac crossings in atomically engineered Co films. These Dirac-like band crossings are fundamentally distinct from a single Dirac point, whose existence and Berry phase characteristics are governed solely by the bulk symmetries. The results highlight the role of dimensionality in determining magnonic band formation, band crossings, and their possible symmetry protection. Furthermore, our discovery of room-temperature Dirac magnons in a thin film enables rigorous testing of the fundamental physics of Dirac bosons within a highly controllable experimental environment. Our results clearly demonstrate that the high-resolution electron scattering techniques, using low-energy electrons, can be used to probe and pinpoint the magnonic states of a thin ferromagnet having a nonzero Berry phase driven not only by the symmetry of the surface itself but also by the symmetry considerations of the layer stacking. The results indicate that Dirac magnons must exist in a vast variety of hcp ferromagnetic 3d films and superlattices, epitaxially grown on nonmagnetic substrates, where the number and sequence of the atomic layers would provide the means for tuning the Berry phase in a well-controlled and layer-by-layer manner. We anticipate that the same symmetry arguments can be used to design magnetic metamaterials, hosting Dirac magnons. To realize Dirac magnons in these systems, one would need to prepare 2D honeycomb-like structures, featuring two distinct magnetic sublattices, similar to the approach used in (*74*). Given that the most important ingredients leading to the formation of Dirac magnons are the symmetry aspects and a nonzero interaction between the two sublattices, both planar and nonplanar structures should host such magnons. All these may open up new horizons for the use of Dirac magnons in the field of magnonics and in all magnon-based logic devices. It is worth emphasizing that the primary outcome of the study is the experimental demonstration of magnonic Dirac crossings in a prototypical, room-temperature ferromagnetic thin film. These results establish a foundational basis for the study of topological bosonic quasiparticles in the terahertz regime within the broader context of fundamental condensed-matter physics. While the implementation of such features in high-frequency architectures remains beyond our present scope, it may represent a promising direction for future research.

## MATERIALS AND METHODS

### Experiments

#### Sample preparation

All the experiments were performed under ultrahigh vacuum conditions with a base pressure of $P≃3\times 10^{-11}$ mbar. Co films were grown on an atomically smooth and clean W(110) substrate. The size of the single crystal used in our study was 5 mm by 8 mm by 0.3 mm. We used a flat W(110) crystal, with a cutting angle better than 0.1°, with respect to the (110) plane of the body-centered cubic structure of tungsten. Before the film growth, the surface of the W(110) substrate was cleaned under ultrahigh vacuum conditions using a cleaning procedure specially developed for refractory metals (*75*). In this procedure, the crystal is flashed at a temperature of about T≃1200 K in an oxygen partial pressure of $P_{{\text{O}}_{2}}≃6\times 10^{-8}\;\text{to}\;9\times 10^{-8}$ mbar to remove the carbon contamination. During each cycle, the flashing power was switched on for 20 s and off for 60 s. In the next step, a couple of high-power flashes (T≃2000 K) were used to desorb the oxygen from the surface. The procedure leads to an atomically clean and well-ordered W(110) surface, as confirmed by our surface structural and chemical analysis by means of low-energy electron diffraction (LEED) and Auger electron spectroscopy, respectively.

The Co layers were deposited onto the clean W(110) surface by means of molecular beam epitaxy at a substrate temperature of about T≃350 to 400 K and with a deposition rate of about 0.23 monolayers per minute (MLs/min). It is well known that for thicknesses below 1 ML, Co grows pseudomorphically on W(110) (*76*). For films with a thickness of above 2 MLs, the structure is hcp with the epitaxial relationship Co(0001)∥W(110). While the Co[$1\bar{1}00$] direction adopts itself along the [$1\bar{1}0$] direction of the W(110) substrate, the large lattice mismatch along the orthogonal direction leads to the formation a superstructure, where five Co atoms correspond to four underlying W atoms (*64*, *76*–*79*). This results in the formation of the so-called floating layer. Because of the finite mean free path of electrons, one clearly observes the presence of such a superstructure in the LEED patterns recorded on samples with a film thickness below 4 MLs. For 15- and 20-ML samples, a perfect hcp structure was observed. In fig. S10A, we show a LEED pattern recorded on a 20-ML sample, taken at an electron energy of 80.2 eV. The corresponding simulated pattern at the same energy is shown in fig. S10B, indicating the perfect hcp structure of the studied samples.

The static magnetic properties of the samples were probed by means of magneto-optical Kerr effect in the longitudinal geometry. Typical magneto-optic Kerr effect hysteresis loops recorded on the studied samples are shown in fig. S10 (C to E). In fig. S10C, the magnetic field was applied along the Co[$1\bar{1}00$] direction (or the W[$1\bar{1}0$] direction). In fig. S10 (D and E), the magnetic field was applied along the Co[$11\bar{2}0$] direction (or the W[100] direction). The results indicate that the Co[$1\bar{1}00$] direction represents an easy magnetization axis.

#### Probing the magnon dispersion relation by means of SPHREELS

Magnons were investigated by means of SPHREELS (*43*–*45*). In SPHREELS, a well-defined monochromatic beam of spin-polarized electrons is scattered from the surface, and the energy and momentum transfer of the electrons is measured at a fixed incident energy (usually both the energy loss and gain of electrons during the scattering event are measured). The spin polarization of the incident electron beam in these experiments was 72±5%. The spectra were recorded for the two possible orientations of the incoming spin-polarized electron beam, i.e., parallel and antiparallel to the scattering plane’s normal vector $\hat{n}$. Before the spectroscopy experiments, a relatively large magnetic field of 0.25 T was applied along $\hat{n}$ to fully magnetize the sample. The spin-polarized spectra were then recorded in the magnetic remanent state, with no magnetic field applied. The scattering cross section was found to be the highest for the incident energy of *E_i_* = 4.0 eV. Therefore, the spectra below ∣Q∣=1.3 Å^−1^ were recorded at this energy. For the spectra at larger ∣Q∣, the incident energy was set to either *E_i_* = 5.0, 7.0, 9.0, or 19.0 eV depending on the wave vector. Considering the surface BZ of hcp(0001), there are two possible geometries to access the $\bar{K}-\bar{M}$ direction (see Fig. 2B). The first approach involves remaining within the first surface BZ and measuring along its edge. The second approach is to extend the measurements beyond the first surface BZ into the second one by measuring along the $\bar{\Gamma }-\bar{K}-\bar{M}$ path. The main challenge here is that at such large wave vectors, the signal count rate becomes very low. Moreover, achieving sufficient momentum transfer requires measurements at higher incident energies. The magnon scattering cross section depends strongly on the incident energy: It is relatively large at lower incident energies (≈4 eV) but decreases considerably at higher energies in a nonmonotonic manner. Consequently, the reduced cross section at higher energies further diminishes the count rate. We adopted the second approach. To enhance the spectrometer’s throughput, the scattering geometry and the pass energies were optimized.

The energy resolution was between 15 and 30 meV, and the spectra were recorded at 300 K. The choice of the resolution was intentional. This optimized the spectrometer’s throughput, enabling the acquisition of high-quality data across a large fraction of the surface BZ within a reasonable time frame. Electrons with their spin parallel to the sample magnetization are referred to as minority electrons, and those with spin polarization antiparallel to the sample magnetization are referred to as majority electrons. Owing to the fact that the total angular momentum during the scattering process must be conserved, a magnon can only be excited when the incident electron’s spin is of minority character. Hence, a peak resulting from the magnon excitations is expected only in the minority spin spectra ($I_{↓}$) and, consequently, a peak in the difference spectra, defined as $I_{↓}-I_{↑}$ (*43*, *44*). The peak is also observable in the spin asymmetry spectra defined as $\left(I_{↓}-I_{↑}\right)/\left(I_{↓}+I_{↑}\right)$.

In the spin-polarized spectra shown in Fig. 2 (C and F) and fig. S4 (A, D, and G), the observed broad features are mainly non–spin-flip in nature (*47*, *80*). The key advantage of SPHREELS is the ability to effectively eliminate the influence of these spin-independent excitations by analyzing the difference spectra. This capability is the essence of performing such dedicated and complex spin-polarized measurements. The magnon wave vector is given by $|Q|=Q_{∥}=k_{i}\text{sin}\theta -k_{f}\text{sin}\left(\theta_{0}-\theta \right)$, where $k_{i}$ ($k_{f}$) is the magnitude of the wave vector of the incident (scattered) electrons, and θ ($\theta_{0}$) is the angle between the incident beam and the sample normal (the scattered beam). To probe magnons with different wave vectors, one may change the incident and scattered angles. In our experiment, this is achieved by rotating the sample about its main axis, i.e., changing θ and keeping $\theta_{0}$ fixed. In a similar way, the constant energy scans are also possible by fixing the energy loss and varying the magnon wave vector. To ensure the reproducibility of the spectra, all measurements were performed on at least three independent films prepared under identical conditions. The data presented reflect independent physical replicates, with consistent spectral features observed across all experimental runs.

To account for the drop in scattering intensity at a large momentum, spectra were normalized to the quasielastic peak at zero energy loss. In addition, where necessary, the high-energy background was adjusted to ensure a consistent comparison across the measured momentum range.

### Theory

#### Calculation of the magnonic band structure and magnonic Bloch spectral function

The magnonic band structure and magnonic Bloch spectral function were calculated by combining the results of ab initio density functional theory (DFT) calculations and adiabatic magnon calculations. The calculations of Co bulk were performed for an infinite system with periodic boundary conditions. In the case of the 20-ML sample, we used the slab calculations. The ab initio DFT calculations provide all the static magnetic properties of the system, e.g., Heisenberg exchange parameters and spin and orbital magnetic moments. In the next step, we calculated the magnonic band structure on the basis of the adiabatic magnon formalism (see below).

Our methodology starts with self-consistent calculations of the electronic structures within the framework of a generalized gradient approximation (GGA + U) of DFT. The Korringa-Kohn-Rostoker Green function formalism is implemented for both three-dimensional bulk magnets and layered structures composed a finite number of layers. The Heisenberg exchange constants were obtained by using the magnetic force theorem (*81*). We use the bulk lattice parameters for constructing the slabs ($a_{1}=a_{2}=a=2.507$ Å and *c* = 4.069 Å; c/a ≈ 1.62). This assumption is justified by our LEED experiments [see, for example, fig. S10 (A and B)]. For k integration, we use a tetrahedral integration method (*82*). A 30 × 30 mesh for self-consistent calculations and a 70 × 70 mesh for the calculations of Heisenberg exchange interactions were used. The slab calculations were performed with a Green function method specially designed for semi-infinite systems (*83*).

The results are in agreement with those of the literature (*38*–*42*). For the slab calculations, we used the lattice parameters of bulk Co. Because of the fact that Co films relax rather quickly on W(110), this is a very good assumption [see also fig. S11 (A and B)]. For the thick Co films studied here, the relativistic effects such as magnetic anisotropy and the antisymmetric interactions have been found to be rather small (*64*, *84*). They are, therefore, neglected in the calculations. Because of the fact that the magnonic band structure of Co spans over several hundreds of milli–electron volts, this is a fairly good assumption. An important consideration is that all adiabatic magnon calculations overestimate the magnonic band structure of Co films (*54*, *55*, *85*–*87*). We have shown previously that the magnonic band structure calculated in this way for flat films agrees both qualitatively and quantitatively with the experimental results only if the effects associated with the many-body correlations have been taken into consideration (*15*, *32*, *51*, *52*, *60*). A practical and less demanding approach to consider such effects is to correct the exchange splitting calculated by the standard DFT. For that, one may rigidly shift the majority bands toward the Fermi level. For Co films, a shift between Δ = 0.8 and 1.0 eV is expected depending on the structure (*51*). In the present study, we introduce a shift of Δ = 0.85 eV, which results in very good agreement with the experimental data. We note that this shift mainly changes the bandwidth of the magnonic band structure and has only minor influences on the shape of the magnon bands. This is illustrated in fig. S11, where we present the results of the magnonic band structure with and without considering the correction for the effects associated with the many-body correlations. Within this framework, one can also calculate the atomic-resolved MBSF and the corresponding atomic-resolved MDOS. In layered structures, it is convenient to project these quantities onto different layers to figure out the contribution of each layer to the total MBSF or MDOS.

#### Calculation of the Berry phase

The Berry phase properties of the observed magnon modes were evaluated using a layer-resolved Heisenberg Hamiltonian. To establish a rigorous connection with our ab initio results, the Hamiltonian is parameterized using site-dependent magnetic moments $m_{i}$ and exchange coupling constants $J_{\mathrm{ij}}$

$$H=-\sum_{i,j}J_{\mathrm{ij}}S_{i}\cdot S_{j}$$ (1)

where $S_{i}$ is the spin operator at the lattice site *i* (with magnitude $S_{i}=m_{i}/g\mu_{B}$). Following a Holstein-Primakoff transformation and subsequent Fourier transform into the 2D momentum space, we solve the bosonic eigenvalue problem to obtain the *N* magnon dispersions $\varepsilon_{n}\left(Q\right)$ and the corresponding eigenvectors $|\psi_{n}\left(Q\right)\rangle$. Here, the mode index *n* ranges from 0 to *N* − 1, consistent with our numerical implementation and the intensity modeling described above. We note that magnon-magnon interactions can theoretically induce energy renormalization or lifetime broadening (*88*). However, these effects were neglected, as the thermal energy scale is much smaller than the magnon bandwidth.

The topological nature of these modes is quantified by the Berry phase, $\phi_{B,n}$, which represents the geometric phase accumulated by the magnon eigenstate $|\psi_{n}\left(Q\right)\rangle$ as it is adiabatically transported along a closed contour C in the 2D surface BZ

$$\phi_{B,n}\left[C\right]=\oint_{C}A_{n}\left(Q\right)\cdot dQ$$ (2)

The integrand $A_{n}\left(Q\right)$ is the Berry connection, defined as

$$A_{n}\left(Q\right)=i\langle \psi_{n}\left(Q\right)|\nabla_{Q}|\psi_{n}\left(Q\right)\rangle$$ (3)

For the specific case of hcp(0001) thin films, our analysis reveals that the nonzero Berry phase is fundamentally rooted in the intersublattice exchange symmetry between the $A_{1}$ and $A_{2}$ sites of the hexagonal lattice. By integrating the Berry connection along closed contours encircling the high-symmetry $\bar{K}$- and ${\bar{K}}^{\prime }$-points, we obtain a quantized Berry phase of $\phi_{B,n}=\pm \pi$. This result confirms the Dirac nature of the magnon crossings and is consistent with observations in related hcp systems (*29*).

A similar formalism was used to calculate the Berry phase of the layered structures. The only difference is that the number of modes *N* is equal to the number of atomic layers of the ferromagnetic film. The results of those analyses are presented in Fig. 5.

We note that the quantized nature of the Berry phase precludes its evolution as a continuous scalar field across the surface BZ. Instead, it is a topological invariant tied to the winding of the complex wave function around the Dirac singularity. The quantized ±π values at the crossing are a direct manifestation of the valley-dependent topological charge, where the sign reflects the chirality of the specific $\bar{K}$ or ${\bar{K}}^{\prime }$ valley.

## Supplementary Materials

### This PDF file includes:

Supplementary Text S1 to S8 Table S1 Figs. S1 to S11 References

## References

[1] P. A. M. Dirac, Quantum mechanics of many-electron systems. Proc. R. Soc. Lond. Ser. A Contain. Pap. Math. Phys. Character 117, 610–624 (1928).

[2] K. S. Novoselov, A. K. Geim, S. V. Morozov, D. Jiang, Y. Zhang, S. V. Dubonos, I. V. Grigorieva, A. A. Firsov, Electric field effect in atomically thin carbon films. Science 306, 666–669 (2004). 15499015 10.1126/science.1102896

[3] A. K. Geim, K. S. Novoselov, The rise of graphene. Nat. Mater. 6, 183–191 (2007). 17330084 10.1038/nmat1849

[4] H. Zhang, C. X. Liu, X. L. Qi, X. Dai, Z. Fang, S. C. Zhang, Topological insulators in Bi_2_Se_3_, Bi_2_Te_3_ and Sb_2_Te_3_ with a single Dirac cone on the surface. Nat. Phys. 5, 438–442 (2009).

[5] M. Z. Hasan, C. L. Kane, Colloquium: Topological insulators. Rev. Mod. Phys. 82, 3045–3067 (2010).

[6] S. M. Young, S. Zaheer, J. C. Y. Teo, C. L. Kane, E. J. Mele, A. M. Rappe, Dirac semimetal in three dimensions. Phys. Rev. Lett. 108, 140405 (2012). 22540776 10.1103/PhysRevLett.108.140405

[7] A. V. Balatsky, I. Vekhter, J.-X. Zhu, Impurity-induced states in conventional and unconventional superconductors. Rev. Mod. Phys. 78, 373–433 (2006).

[8] T. Wehling, A. Black-Schaffer, A. Balatsky, Dirac materials. Adv. Phys. 63, 1–76 (2014).

[9] X. Chen, Y. Liu, P. Liu, Y. Yu, J. Ren, J. Li, A. Zhang, Q. Liu, Unconventional magnons in collinear magnets dictated by spin space groups. Nature 640, 349–354 (2025). 40074910 10.1038/s41586-025-08715-7 PMC11981943

[10] L. Lu, J. D. Joannopoulos, M. Soljacic, Topological photonics. Nat. Photonics 8, 821–829 (2014).

[11] Y. Liu, Y. Xu, W. Duan, Berry phase and topological effects of phonons. Natl. Sci. Rev. 5, 314–316 (2017).

[12] Y. Liu, X. Chen, Y. Xu, Topological phononics: From fundamental models to real materials. Adv. Funct. Mater. 30, 1904784 (2019).

[13] T. Ozawa, H. M. Price, A. Amo, N. Goldman, M. Hafezi, L. Lu, M. C. Rechtsman, D. Schuster, J. Simon, O. Zilberberg, I. Carusotto, Topological photonics. Rev. Mod. Phys. 91, 015006 (2019).

[14] K. Zakeri, Terahertz magnonics: Feasibility of using terahertz magnons for information processing. Phys. C Supercond. Appl. 549, 164–170 (2018).

[15] K. Zakeri, Magnonic crystals: Towards terahertz frequencies. J. Phys. Condens. Matter 32, 363001 (2020). 10.1088/1361-648X/ab88f2 32289765

[16] H. Yuan, Y. Cao, A. Kamra, R. A. Duine, P. Yan, Quantum magnonics: When magnon spintronics meets quantum information science. Phys. Rep. 965, 1–74 (2022).

[17] A. Mook, J. Henk, I. Mertig, Tunable magnon Weyl points in ferromagnetic pyrochlores. Phys. Rev. Lett. 117, 157204 (2016). 27768368 10.1103/PhysRevLett.117.157204

[18] S. Bao, J. Wang, W. Wang, Z. Cai, S. Li, Z. Ma, D. Wang, K. Ran, Z. Y. Dong, D. L. Abernathy, S. L. Yu, X. Wan, J. X. Li, J. Wen, Discovery of coexisting Dirac and triply degenerate magnons in a three-dimensional antiferromagnet. Nat. Commun. 9, 2591 (2018). 29968725 10.1038/s41467-018-05054-2 PMC6030121

[19] W. Yao, C. Li, L. Wang, S. Xue, Y. Dan, K. Iida, K. Kamazawa, K. Li, C. Fang, Y. Li, Topological spin excitations in a three-dimensional antiferromagnet. Nat. Phys. 14, 1011–1015 (2018).

[20] Y. Jin, R. Wang, H. Xu, Recipe for Dirac phonon states with a quantized valley Berry phase in two-dimensional hexagonal lattices. Nano Lett. 18, 7755–7760 (2018). 30456958 10.1021/acs.nanolett.8b03492

[21] L. Chen, J. H. Chung, B. Gao, T. Chen, M. B. Stone, A. I. Kolesnikov, Q. Huang, P. Dai, Topological spin excitations in honeycomb ferromagnet CrI_3_. Phys. Rev. X 8, 041028 (2018). 10.1103/PhysRevX.8.041028 PMC11194793 38915421

[22] B. Yuan, I. Khait, G. J. Shu, F. C. Chou, M. B. Stone, J. P. Clancy, A. Paramekanti, Y. J. Kim, Dirac magnons in a honeycomb lattice quantum *XY* magnet CoTiO_3_. Phys. Rev. X 10, 011062 (2020).

[23] M. Elliot, P. A. McClarty, D. Prabhakaran, R. D. Johnson, H. C. Walker, P. Manuel, R. Coldea, Order-by-disorder from bond-dependent exchange and intensity signature of nodal quasiparticles in a honeycomb cobaltate. Nat. Commun. 12, 3936 (2021). 34168125 10.1038/s41467-021-23851-0 PMC8225819

[24] J. A. Schneeloch, Y. Tao, Y. Cheng, L. Daemen, G. Xu, Q. Zhang, D. Louca, Gapless Dirac magnons in CrCl_3_. NPJ Quantum Mater. 7, 66 (2022).

[25] S.-H. Do, K. Kaneko, R. Kajimoto, K. Kamazawa, M. B. Stone, J. Y. Y. Lin, S. Itoh, T. Masuda, G. D. Samolyuk, Damped Dirac magnon in the metallic kagome antiferromagnet FeSn. Phys. Rev. B 105, l180403 (2022).

[26] S. Nikitin, B. Fåk, K. W. Krämer, T. Fennell, B. Normand, A. M. Läuchli, C. Rüegg, Thermal evolution of Dirac magnons in the honeycomb ferromagnet CrBr_3_. Phys. Rev. Lett. 129, 127201 (2022). 36179160 10.1103/PhysRevLett.129.127201

[27] M. dos Santos Dias, N. Biniskos, F. J. dos Santos, K. Schmalzl, J. Persson, F. Bourdarot, N. Marzari, S. Blügel, T. Brückel, S. Lounis, Topological magnons driven by the Dzyaloshinskii-Moriya interaction in the centrosymmetric ferromagnet Mn_5_Ge_3_. Nat. Commun. 14, 7321 (2023). 37951946 10.1038/s41467-023-43042-3 PMC10640582

[28] M. Terilli, X. Jia, X. Liu, P. Laurell, A. M. Nedić, Y. Chang, T. C. Wu, H. Chen, H. Li, M. H. Upton, J. Kim, J. W. Kim, P. J. Ryan, C. Nelson, J. Zhou, M. Kareev, W. Hu, J. H. Pixley, G. A. Fiete, Y. Cao, J. Chakhalian, Spectrally sharp magnetic excitations above the critical temperature in a frustrated Weyl semimetal. Nat. Commun. 16, 6576 (2025). 40675956 10.1038/s41467-025-61752-8 PMC12271449

[29] A. Scheie, P. Laurell, P. A. McClarty, G. E. Granroth, M. B. Stone, R. Moessner, S. E. Nagler, Dirac magnons, nodal lines, and nodal plane in elemental gadolinium. Phys. Rev. Lett. 128, 097201 (2022). 35302826 10.1103/PhysRevLett.128.097201

[30] A. Scheie, P. Laurell, P. A. McClarty, G. E. Granroth, M. B. Stone, R. Moessner, S. E. Nagler, Spin-exchange Hamiltonian and topological degeneracies in elemental gadolinium. Phys. Rev. B 105, 104402 (2022). 10.1103/PhysRevLett.128.097201 35302826

[31] G. Sundaram, Q. Niu, Wave-packet dynamics in slowly perturbed crystals: Gradient corrections and Berry-phase effects. Phys. Rev. B 59, 14915–14925 (1999).

[32] K. Zakeri, H. Qin, A. Ernst, Unconventional magnonic surface and interface states in layered ferromagnets. Commun. Phys. 4, 18 (2021).

[33] P. A. Lindgard, B. N. Harmon, A. J. Freeman, Theoretical magnon dispersion curves for Gd. Phys. Rev. Lett. 35, 383–386 (1975).

[34] J. Jensen, A. R. Mackintosh, *Rare Earth Magnetism: Structures and Excitations*, vol. 81 of *International Series of Monographs on Physics* (Clarendon Press, Oxford, UK) (1991).

[35] P. Buczek, A. Ernst, L. M. Sandratskii, Different dimensionality trends in the Landau damping of magnons in iron, cobalt, and nickel: Time-dependent density functional study. Phys. Rev. B 84, 174418 (2011).

[36] T. Skovhus, T. Olsen, Dynamic transverse magnetic susceptibility in the projector augmented-wave method: Application to Fe, Ni, and Co. Phys. Rev. B 103, 245110 (2021).

[37] K. Zakeri, A. Ernst, Dimensionality-driven terahertz Dirac magnons in layered *3d* ferromagnets. Nano Lett. 26, 2660–2665 (2026). 41671471 10.1021/acs.nanolett.5c06192

[38] S. V. Halilov, H. Eschrig, A. Y. Perlov, P. M. Oppeneer, Adiabatic spin dynamics from spin-density-functional theory: Application to Fe, Co, and Ni. Phys. Rev. B 58, 293–302 (1998).

[39] M. Pajda, J. Kudrnovský, I. Turek, V. Drchal, P. Bruno, Ab initio calculations of exchange interactions, spin-wave stiffness constants, and Curie temperatures of Fe, Co, and Ni. Phys. Rev. B 64, 174402 (2001).

[40] I. Turek, J. Kudrnovsky, V. Drchal, P. Bruno, Exchange interactions, spin waves, and transition temperatures in itinerant magnets. Philos. Mag. 86, 1713–1752 (2006).

[41] H. Okumura, K. Sato, T. Kotani, Spin-wave dispersion of 3*d* ferromagnets based on quasiparticle self-consistent *GW* calculations. Phys. Rev. B 100, 054419 (2019).

[42] H. Ebert, S. Mankovsky, S. Wimmer, *Electronic Structure: Metals and Insulators* (Springer International Publishing, 2021), pp. 1–73.

[43] R. Vollmer, M. Etzkorn, P. S. A. Kumar, H. Ibach, J. Kirschner, Spin-polarized electron energy loss spectroscopy of high energy, large wave vector spin waves in ultrathin fcc Co films on Cu(001). Phys. Rev. Lett. 91, 147201 (2003). 14611549 10.1103/PhysRevLett.91.147201

[44] K. Zakeri, J. Kirschner, *Probing Magnons by Spin-Polarized Electrons*, vol. 125 of *Topics in Applied Physics Magnonics from Fundamentals to Applications* (Springer, Berlin, Heidelberg, 2013), chap. 7, pp. 84–99.

[45] K. Zakeri, Elementary spin excitations in ultrathin itinerant magnets. Phys. Rep. 545, 47–93 (2014).

[46] K. Zakeri, D. Rau, J. Jandke, F. Yang, W. Wulfhekel, C. Berthod, Direct probing of a large spin-orbit coupling in the FeSe superconducting monolayer on STO. ACS Nano 17, 9575–9585 (2023). 37155694 10.1021/acsnano.3c02876

[47] J. Kirschner, Direct and exchange contributions in inelastic scattering of spin-polarized electrons from iron. Phys. Rev. Lett. 55, 973–976 (1985). 10032497 10.1103/PhysRevLett.55.973

[48] K. Zakeri, T. H. Chuang, A. Ernst, L. M. Sandratskii, P. Buczek, H. J. Qin, Y. Zhang, J. Kirschner, Direct probing of the exchange interaction at buried interfaces. Nat. Nanotechnol. 8, 853–858 (2013). 24056902 10.1038/nnano.2013.188

[49] K. Zakeri, Y. Zhang, J. Kirschner, Surface magnons probed by spin-polarized electron energy loss spectroscopy. J. Electron. Spectros. Relat. Phenomena 189, 157–163 (2013).

[50] E. Michel, H. Ibach, C. M. Schneider, Spin waves in ultrathin hexagonal cobalt films on W(110), Cu(111), and Au(111) surfaces. Phys. Rev. B 92, 024407 (2015).

[51] Y.-J. Chen, K. Zakeri, A. Ernst, H. J. Qin, Y. Meng, J. Kirschner, Group velocity engineering of confined ultrafast magnons. Phys. Rev. Lett. 119, 267201 (2017). 29328716 10.1103/PhysRevLett.119.267201

[52] H. J. Qin, S. Tsurkan, A. Ernst, K. Zakeri, Experimental realization of atomic-scale magnonic crystals. Phys. Rev. Lett. 123, 257202 (2019). 31922781 10.1103/PhysRevLett.123.257202

[53] K. Zakeri, A. Ernst, Generation and propagation of ultrafast terahertz magnons in atomically architectured nanomagnets. Nano Lett. 24, 9528–9534 (2024). 38899856 10.1021/acs.nanolett.4c01982

[54] L. Bergqvist, A. Taroni, A. Bergman, C. Etz, O. Eriksson, Atomistic spin dynamics of low-dimensional magnets. Phys. Rev. B 87, 144401 (2013).

[55] C. Etz, L. Bergqvist, A. Bergman, A. Taroni, O. Eriksson, Atomistic spin dynamics and surface magnons. J. Phys. Condens. Matter 27, 243202 (2015). 26030259 10.1088/0953-8984/27/24/243202

[56] A. T. Costa, R. B. Muniz, D. L. Mills, Spin waves and their damping in itinerant ultrathin ferromagnets: Intermediate wave vectors. Phys. Rev. B 74, 214403 (2006).

[57] D. L. Mills, *Spin Waves: History and a Summary of Recent Developments*, vol. 1 of *Handbook of Magnetism and Advanced Magnetic Materials* (Wiley & Sons Ltd., 2007), chap. Fundamentals and Theory, pp. 247–282.

[58] Y. Zhang, T.-H. Chuang, K. Zakeri, J. Kirschner, Relaxation time of terahertz magnons excited at ferromagnetic surfaces. Phys. Rev. Lett. 109, 087203 (2012). 23002772 10.1103/PhysRevLett.109.087203

[59] K. Zakeri, A. Hjelt, I. Maznichenko, P. Buczek, A. Ernst, Nonlinear decay of quantum confined magnons in itinerant ferromagnets. Phys. Rev. Lett. 126, 177203 (2021). 33988456 10.1103/PhysRevLett.126.177203

[60] S. Paischer, D. Eilmsteiner, I. Maznichenko, N. Buczek, K. Zakeri, A. Ernst, P. A. Buczek, Correlations, disorder, and multimagnon processes in terahertz spin dynamics of magnetic nanostructures: A first-principles investigation. Phys. Rev. B 109, l220405 (2024).

[61] R. Matsumoto, S. Murakami, Theoretical prediction of a rotating magnon wave packet in ferromagnets. Phys. Rev. Lett. 106, 197202 (2011). 21668195 10.1103/PhysRevLett.106.197202

[62] K. Zakeri, Y. Zhang, J. Prokop, T. H. Chuang, N. Sakr, W. X. Tang, J. Kirschner, Asymmetric spin-wave dispersion on Fe(110): Direct evidence of the Dzyaloshinskii-Moriya interaction. Phys. Rev. Lett. 104, 137203 (2010). 20481909 10.1103/PhysRevLett.104.137203

[63] K. Zakeri, Y. Zhang, J. Prokop, T.-H. Chuang, W. X. Tang, J. Kirschner, Magnon excitations in ultrathin Fe layers: The influence of the Dzyaloshinskii-Moriya interaction. J. Phys. Conf. Ser. 303, 012004 (2011).

[64] S. Tsurkan, K. Zakeri, Giant Dzyaloshinskii-Moriya interaction in epitaxial Co/Fe bilayers with *C*_2*v*_ symmetry. Phys. Rev. B 102, 060406 (2020).

[65] K. Zakeri, A. von Faber, Giant spin-orbit induced magnon nonreciprocity in ultrathin ferromagnets. Phys. Rev. Lett. 132, 126702 (2024). 38579230 10.1103/PhysRevLett.132.126702

[66] K. Zakeri, A. von Faber, S. Mankovsky, H. Ebert, Unraveling the complexity of the Dzyaloshinskii-Moriya interaction in layered magnets: The full magnitude and chirality control. Adv. Mater. 37, e2500152 (2025). 40589322 10.1002/adma.202500152 PMC12412010

[67] H. J. Qin, K. Zakeri, A. Ernst, L. M. Sandratskii, P. Buczek, A. Marmodoro, T.-H. Chuang, Y. Zhang, J. Kirschner, Long-living terahertz magnons in ultrathin metallic ferromagnets. Nat. Commun. 6, 6126 (2015). 25625857 10.1038/ncomms7126

[68] H. J. Qin, K. Zakeri, A. Ernst, J. Kirschner, Temperature dependence of magnetic excitations: Terahertz magnons above the Curie temperature. Phys. Rev. Lett. 118, 127203 (2017). 28388202 10.1103/PhysRevLett.118.127203

[69] P. A. McClarty, J. G. Rau, Non-Hermitian topology of spontaneous magnon decay. Phys. Rev. B 100, 100405 (2019).

[70] A. B. Schmidt, M. Pickel, M. Donath, P. Buczek, A. Ernst, V. P. Zhukov, P. M. Echenique, L. M. Sandratskii, E. V. Chulkov, M. Weinelt, Ultrafast magnon generation in an Fe film on Cu(100). Phys. Rev. Lett. 105, 197401 (2010). 21231194 10.1103/PhysRevLett.105.197401

[71] K. Zakeri, J. Wettstein, C. Sürgers, Generation of spin-polarized hot electrons at topological insulators surfaces by scattering from collective charge excitations. Commun. Phys. 4, 225 (2021).

[72] T. Balashov, A. F. Takács, W. Wulfhekel, J. Kirschner, Magnon excitation with spin-polarized scanning tunneling microscopy. Phys. Rev. Lett. 97, 187201 (2006). 17155571 10.1103/PhysRevLett.97.187201

[73] D. Kepaptsoglou, J. Á. Castellanos-Reyes, A. Kerrigan, J. Alves do Nascimento, P. M. Zeiger, K. El Hajraoui, J. C. Idrobo, B. G. Mendis, A. Bergman, V. K. Lazarov, J. Rusz, Q. M. Ramasse, Magnon spectroscopy in the electron microscope. Nature 644, 83–88 (2025). 40702193 10.1038/s41586-025-09318-y PMC12328233

[74] J. Chen, M. Madami, G. Gubbiotti, H. Yu, Magnon confinement in a nanomagnonic waveguide by a magnetic Moiré superlattice. Appl. Phys. Lett. 125, 162403 (2024).

[75] K. Zakeri, T. Peixoto, Y. Zhang, J. Prokop, J. Kirschner, On the preparation of clean tungsten single crystals. Surf. Sci. 604, L1–L3 (2010).

[76] M. Pratzer, H. J. Elmers, M. Getzlaff, Heteroepitaxial growth of Co on W(110) investigated by scanning tunneling microscopy. Phys. Rev. B 67, 153405 (2003).

[77] H. Fritzsche, J. Kohlhepp, U. Gradmann, Epitaxial strain and magnetic anisotropy in ultrathin Co films on W(110). Phys. Rev. B 51, 15933–15941 (1995). 10.1103/physrevb.51.15933 9978572

[78] M. Etzkorn, P. S. Anil Kumar, W. Tang, Y. Zhang, J. Kirschner, High-wave-vector spin waves in ultrathin Co films on W(110). Phys. Rev. B 72, 184420 (2005).

[79] M. Etzkorn, P. S. A. Kumar, J. Kirschner, *High-energy Surface Spin Waves Studied by Spin-polarized Electron Energy Loss Spectroscopy*, vol. 3 of *Handbook of Magnetism and Advanced Magnetic Materials* (Wiley & Sons Ltd., 2007), chap. Spin-polarized electron spectroscopies, pp. 1658–1684.

[80] K. Zakeri, C. Berthod, Theory of spin-polarized high-resolution electron energy loss spectroscopy from nonmagnetic surfaces with a large spin-orbit coupling. Phys. Rev. B 106, 235117 (2022).

[81] A. I. Liechtenstein, M. I. Katsnelson, V. P. Antropov, V. A. Gubanov, Local spin density functional approach to the theory of exchange interactions in ferromagnetic metals and alloys. J. Magn. Magn. Mater. 67, 65–74 (1987).

[82] P. E. Blöchl, O. Jepsen, O. K. Andersen, Improved tetrahedron method for Brillouin-zone integrations. Phys. Rev. B 49, 16223–16233 (1994). 10.1103/physrevb.49.16223 10010769

[83] L. Szunyogh, B. Újfalussy, P. Weinberger, J. Kollár, Self-consistent localized KKR scheme for surfaces and interfaces. Phys. Rev. B 49, 2721–2729 (1994). 10.1103/physrevb.49.2721 10011105

[84] K. Zakeri, A. Marmodoro, A. von Faber, S. Mankovsky, H. Ebert, Chirality-inverted Dzyaloshinskii-Moriya interaction. Phys. Rev. B 108, l100403 (2023).

[85] F. J. dos Santos, M. dos Santos Dias, S. Lounis, First-principles investigation of spin-wave dispersions in surface-reconstructed Co thin films on W(110). Phys. Rev. B 95, 134408 (2017).

[86] A. T. Costa Jr., R. B. Muniz, D. L. Mills, Theory of spin waves in ultrathin ferromagnetic films: The case of Co on Cu(100). Phys. Rev. B 69, 064413 (2004).

[87] A. T. Costa, R. B. Muniz, D. L. Mills, Theory of large-wave-vector spin waves in ultrathin ferromagnetic films: Sensitivity to electronic structure. Phys. Rev. B 70, 054406 (2004).

[88] S. S. Pershoguba, S. Banerjee, J. C. Lashley, J. Park, H. Ågren, G. Aeppli, A. V. Balatsky, Dirac magnons in honeycomb ferromagnets. Phys. Rev. X 8, 011010 (2018).

[89] T.-H. Chuang, K. Zakeri, A. Ernst, L. M. Sandratskii, P. Buczek, Y. Zhang, H. J. Qin, W. Adeagbo, W. Hergert, J. Kirschner, Impact of atomic structure on the magnon dispersion relation: A comparison between Fe(111)/Au/W(110) and Fe(110)/W(110). Phys. Rev. Lett. 109, 207201 (2012). 23215520 10.1103/PhysRevLett.109.207201

[90] M. A. Ruderman, C. Kittel, Indirect exchange coupling of nuclear magnetic moments by conduction electrons. Phys. Rev. 96, 99–102 (1954).

[91] T. Kasuya, A theory of metallic ferro- and antiferromagnetism on Zeners model. Prog. Theor. Phys. 16, 45–57 (1956).

[92] K. Yosida, Magnetic properties of Cu-Mn alloys. Phys. Rev. 106, 893–898 (1957).

[93] K. Zakeri, J. Prokop, Y. Zhang, J. Kirschner, Magnetic excitations in ultrathin magnetic films: Temperature effects. Surf. Sci. 630, 311–316 (2014).

[94] J. Prokop, W. X. Tang, Y. Zhang, I. Tudosa, T. R. F. Peixoto, K. Zakeri, J. Kirschner, Magnons in a ferromagnetic monolayer. Phys. Rev. Lett. 102, 177206 (2009). 19518825 10.1103/PhysRevLett.102.177206

[95] K. Zakeri, A. von Faber, A. Ernst, Magnons and fundamental magnetic interactions in a ferromagnetic monolayer: The case of the Ni monolayer. Phys. Rev. B 109, l180406 (2024).

