# Supplementary material for: Dirac magnons in a thin elemental itinerant ferromagnet
Source: Sci Adv. 2026 Jul 15;12(29):eaed9835. doi: 10.1126/sciadv.aed9835 (PMC13371892; doi:10.1126/sciadv.aed9835)
Supplement: Supplementary file 1 — Supplementary Text S1 to S8 Table S1 Figs. S1 to S11 References [file sciadv.aed9835_sm.pdf]

Science Advances
AAAS

Supplementary Materials for

Dirac magnons in a thin elemental itinerant ferromagnet

Khalil Zakeri *et al.*

Corresponding author: Khalil Zakeri, khalil.zakeri@kit.edu

Sci. Adv. **12**, eaed9835 (2026)
DOI: 10.1126/sciadv.aed9835

This PDF file includes:

Supplementary Text S1 to S8
Table S1
Figs. S1 to S11
References

□ Supplementary Text

S1. Possibility of Dirac magnon formation in layered hcp ferromagnetic structures

To investigate the potential formation of Dirac magnons in hcp films with various surface orientations, we conducted a symmetry-based analysis. The presence of a Dirac point in bulk hcp ferromagnets is governed by the bulk symmetry and the little group at high-symmetry points of the Brillouin zone. Specifically, the K–point is of particular interest, as it hosts symmetry-protected Dirac magnons.

Bulk vs. surface symmetry

The relevant symmetry group for analyzing the symmetry-driven protection of magnon bands is the magnetic space group (also referred to as the spin space group). However, for ferromagnets lacking antisymmetric exchange interactions (such as the Dzyaloshinskii–Moriya interaction), the magnetic space group is intimately related to the crystallographic space group. Therefore, the analysis of the crystallographic space group is often sufficient for simplicity and convenience. The crystal structure of cobalt at ambient conditions is hcp, with a two-atom basis located at positions $(0,0,0)$ and $\left(\frac{2}{3},\frac{1}{3},\frac{1}{2}\right)$. The space group is $P6_3/mmc$ (No. 194), and the corresponding point group is D_{6h} $(6/mmm)$. This detailed symmetry characterization is essential for analyzing band degeneracies and excitation spectra in hcp Co(0001) slabs.

In the context of symmetry analysis for electronic, phononic, or magnonic states, the small (or little) groups at high-symmetry points in the Brillouin zone are important. These symmetries are as follows: Γ and A points retain the full D_{6h} symmetry, K and H points have D_{3h} symmetry, M and L points have D_{2h} symmetry, and along high-symmetry lines, the Λ (Γ–A) line has C_{6v} symmetry, the Σ (Γ–M) line has C_{2v} symmetry, and the P (K–H) line has C_{3v} symmetry. Once a slab composed of a few layers of Co(0001) is formed, the overall symmetry is lowered to C_{3v}. The small (or little) groups at high-symmetry $\bar{K}$–point in the surface Brillouin zone remains C_{3v}.

When considering the hcp(0001) surface, each individual layer exhibits a two-dimensional hexagonal lattice. However, due to the specific layer stacking sequence ($A_1A_2A_1A_2$...), the symmetry of the surface termination is reduced to the C_{3v} point group. This reduction preserves the three-fold rotational symmetry (C_3) around the surface normal and three vertical mirror planes (σ_v). In contrast, analyzing the prismatic surfaces, such as the $(10\bar{1}0)$ and $(11\bar{2}0)$ facets, reveals a further reduction in symmetry. Since these surfaces represent a cut across the stacking direction, the in-plane symmetry is reduced to the C_{2v} point group, and the three-fold rotational symmetry is lost.

The preceding analysis physically implies that Dirac magnon protection is preserved exclusively

on surfaces parallel to the basal plane, as they maintain the required three-fold rotational symmetry (C_3). The expected Berry phases at the high-symmetry points of various hcp surfaces are summarized in Table S1. This indicates that a nonzero Berry phase is expected at the $\bar{K}$-point of the hcp(0001) surface Brillouin zone.

S2. Magnon modes observed in the spectra and their thickness dependence

General considerations

Generally, a ferromagnetic film of N layers exhibits N magnon modes due to quantum confinement along the growth axis, provided each layer contains a single magnetic sublattice (32). Within this framework, one can associate a quantum number $n = 0, 1, 2, \ldots, N-1$ to each magnon mode. Except for the $n = 0$ (uniform) mode, the energy of each mode at the $\bar{\Gamma}$-point depends strongly on the film thickness N. The difference spectra measured by SPHREELS include contributions from both these surface and bulk-like modes.

To understand the physical origin of these measured spectra, we consider the generalized layer-resolved susceptibility matrix, $\chi_{ll'}(\boldsymbol{Q}, \omega)$, which describes the magnetic response of atomic layer l to a transverse magnetic perturbation applied in layer l'. In itinerant electron systems, the calculation of $\chi_{ll'}(\boldsymbol{Q}, \omega)$ naturally treats collective spin-wave excitations and single-particle Stoner excitations on an equal footing (35, 36, 45, 48, 67, 86, 87). The coupling between the collective magnons and the electronic Stoner continuum leads to finite quasiparticle lifetimes, known as Landau damping. Consequently, the dynamic susceptibility develops poles in the complex frequency plane corresponding to these damped excitations.

Near these resonances, the susceptibility matrix can be expressed using a spectral pole expansion. Because the susceptibility matrix is generally non-Hermitian in the presence of damping, the expansion involves biorthogonal left and right eigenvectors. Denoting the right eigenvectors by $\psi_l^{(n)}(\boldsymbol{Q})$ and the corresponding left eigenvectors by $\phi_l^{(n)}(\boldsymbol{Q})$, the matrix elements are given by:

$$\chi_{ll'}(\boldsymbol{Q}, \omega) \approx \sum_{n=0}^{N-1} \frac{\psi_l^{(n)}(\boldsymbol{Q})\, \phi_{l'}^{(n)}(\boldsymbol{Q})}{\omega - \varepsilon_n(\boldsymbol{Q})/\hbar + i\Gamma_n(\boldsymbol{Q}, \omega)}, \tag{S1}$$

where $\varepsilon_n(\boldsymbol{Q})$ is the magnon eigenenergy and $\Gamma_n(\boldsymbol{Q}, \omega)$ represents the intrinsic damping resulting from the coupling to the Stoner continuum. The eigenvectors satisfy the biorthogonality relation $\sum_l \phi_l^{(n)}(\boldsymbol{Q})\, \psi_l^{(m)}(\boldsymbol{Q}) = \delta_{nm}$.

In SPHREELS, the probe does not couple equally to all layers. The incident and scattered fields (effective interaction potentials) decay into the bulk, imposing a depth-dependent attenuation factor $\mathcal{A}_l(\theta)$. The measured macroscopic susceptibility corresponds to a projection of the intrinsic response onto this probe penetration profile: $\chi(\boldsymbol{Q}, \omega) = \sum_{l,l'} \mathcal{A}_l(\theta)\, \chi_{ll'}(\boldsymbol{Q}, \omega)\, \mathcal{A}_{l'}(\theta)$. Substituting the pole expansion factorizes the layer summations:

$$\chi(\boldsymbol{Q}, \omega) = \sum_{n=0}^{N-1} \frac{\left(\sum_{l=1}^{N} \mathcal{A}_l(\theta)\psi_l^{(n)}(\boldsymbol{Q})\right)\left(\sum_{l'=1}^{N} \mathcal{A}_{l'}(\theta)\phi_{l'}^{(n)}(\boldsymbol{Q})\right)}{\omega - \varepsilon_n(\boldsymbol{Q})/\hbar + i\Gamma_n(\boldsymbol{Q}, \omega)}. \tag{S2}$$

Assuming the susceptibility matrix is approximately Hermitian (as is typical for weak damping), the eigenvectors become complex conjugates, $\phi_l^{(n)} \approx [\psi_l^{(n)}]^*$. The experimental intensity $I(\boldsymbol{Q}, \omega)$ is proportional to the dissipative part of this effective susceptibility, $-\mathrm{Im}\,\chi(\boldsymbol{Q}, \omega)$, yielding:

$$I(\boldsymbol{Q}, \omega) \propto \sum_{n=0}^{N-1} \left| \sum_{l=1}^{N} \mathcal{A}_l(\theta) \psi_l^{(n)}(\boldsymbol{Q}) \right|^2 \frac{\Gamma_n(\boldsymbol{Q}, \omega)}{[\omega - \varepsilon_n(\boldsymbol{Q})/\hbar]^2 + \Gamma_n^2(\boldsymbol{Q}, \omega)}. \tag{S3}$$

The term $|\ldots|^2$ represents the effective spectral weight of the n-th mode. In practice, this intrinsic Lorentzian response is convoluted with a Gaussian instrumental resolution (Δ_G), resulting in the Voigt profiles used to fit the experimental data. It has been demonstrated that such a convolution reproduces the experimental spectra remarkably well if the film is only a few atomic layers thick e.g., one or two atomic layers (45, 67).

Justification of the two-peak description

For a film of 20 atomic layers, the spectrum theoretically supports 20 discrete quantization modes. Since these modes are localized in different layers and are not excited equally by low-energy electrons, it would be unrealistic to attempt a full fit using 20 Voigt functions, which would necessitate handling approximately $20 \times 3 = 60$ independent fitting parameters, even when treating the effective spectral weight as a single variable. The most important characteristic of magnons in Co hcp(0001) films is the classification of these modes into two distinct groups, which correspond to the two sublattices of the hcp structure. These two groups form the pairs of magnon bands that give rise to the Dirac cones at the $\bar{K}$ point. Given that the dominant contributions to these two magnon groups originate from the two topmost layers, simplifying the analysis by considering two peak functions in momentum space to estimate the magnon energies is physically reasonable.

Clarification of analysis scope

It is crucial to emphasize that we do not aim to perform a full quantitative fit to the experimental data. The peak functions presented in Figure 2 of the manuscript are intended only to qualitatively mimic the main experimental features and should therefore not be regarded as a complete description of the spectra and may be considered as a guide to the eye. Consequently, this analysis does not yield meaningful quantitative estimates of experimental broadening or the lifetime of the observed modes. However, these limitations do not influence the main conclusions of the manuscript.

In Figure S1 we present the difference spectra recorded at the wavevector of $Q = 0.6$ Å^{-1} on samples with different thicknesses. For the sake of better comparison each spectrum is normalized to its maximum. Since the number and the position of the bulk modes depend strongly on the number of Co layers, the thicker the layer the more dense are the bulk modes in the momentum–energy space.

In the case of the thin samples (thicknesses of 2 and 3 ML) the observed sharp signal in the difference spectra can, therefore, be associated with the acoustic surface magnon mode. In the case of the sample with the thickness of 15 and 20 ML one observes the acoustic surface mode very similar to that of the thinner samples. In addition to that one clearly recognizes a tail and several satellites, appearing after this mode. All these features can be attributed to the bulk modes. Note that due to the presence of the Landau damping all these modes possess a finite lifetime broadening. Since the Landau damping can, in principle, exhibit a nonlinear behavior, it is difficult to predict the damping of each mode (59). However, as a rule of thumb one may assume that the peak broadening scales with the energy. In addition, owing to the surface sensitivity of SPHREELS the contribution of the inner layers to the spectra is much weaker than that of the surface layer. Hence, the bulk modes appear only as a tail after the surface mode, instead of sharp peaks, as seen in Figure S1.

The arguments discussed above are also valid for the α_2 modes. In this case the so-called optical surface mode is the main feature. This mode is mainly localized in the second topmost layer (the layer composed of A_2 sublattices). The broad spectrum, is caused by the large Landau damping of this class of magnon modes. It is very useful to analyze the intensity map in the momentum-energy space, instead of trying to resolve the individual modes within α_1 and α_2. Since all the individual modes within each class originate from the same physical principles, investigation of the behavior of the whole class is sufficient to understand the physics hidden behind. Therefore, the peak functions shown by the solid curves in Figure S1 and Figure 2 of the manuscript are not meant to fully reproduce the experimental data. They shall rather show the energetic position of the α_1 and α_2 modes (see the discussion below).

As a side remark, the reduced visibility of the higher-energy modes in the asymmetry spectra shown in Figures 2(**E**) and (**H**) can be traced back directly to its mathematical definition: Asymmetry = $\frac{I_\downarrow - I_\uparrow}{I_\downarrow + I_\uparrow}$, where $I_\downarrow$ and $I_\uparrow$ denote the spin-polarized spectra corresponding to the two possible orientations of the spin polarization vector (parallel and antiparallel to the ground-state magnetization). Since the total intensity ($I_\downarrow + I_\uparrow$) appears in the denominator, it is expected that the magnon peaks are less pronounced in the asymmetry spectra. At higher energy losses, the total intensity in the denominator remains significant due to the broad electronic background and multiple scattering events. This is clearly visible in our spin-polarized spectra shown in Figures 2(**C**) and (**F**), where the large, broad background centered around an energy loss of $\approx$ 500 meV arises from these processes. The various contributions to inelastic electron scattering from magnetic surfaces were investigated in the seminal work by J. Kirschner (47) and have also been recently discussed in Ref. (80). It has been demonstrated that, in addition to spin-flip excitations, there is a substantial contribution from non-spin-flip processes.

This is to be expected, as incident electrons participate in diverse inelastic scattering channels. While some involve spin-flip exchange processes (such as Stoner pair creation), many single-particle electron-hole excitations are inherently non-spin-flip in nature.

Consequently, even though the magnon signal ($I_{\downarrow} - I_{\uparrow}$) is well-resolved in the difference spectrum, its relative weight in the asymmetry is mathematically suppressed by the total (sum) intensity. This does not imply an inconsistency. It rather reflects the different physical information emphasized by each representation. The modes are physically present in both, but the difference spectrum provides much higher contrast for identifying individual magnon branches.

The fact that the asymmetry remains large and positive in this energy range is a "smoking gun" for the magnetic nature of the features observed in the difference spectra. While the peaks in the asymmetry spectra appear as smaller features ("bumps"), their presence is unambiguous.

Further consideration for analysis of the difference spectra

A fully quantitative theory accounting for spin-dependent inelastic electron scattering from magnetic surfaces remains a formidable challenge in $3d$ transition metals. However, the observed spectral distribution can be rigorously justified by considering the surface sensitivity of the probe, layer-dependent damping, and the influence of the underlying electronic band structure.

In the following we briefly discuss the main considerations for a more detailed analysis of the difference spectra:

Probing depth and the inelastic mean free path (IMFP): In the energy range of our SPHREELS experiments ($E_i \approx$ 4–20 eV), the IMFP in transition metal ferromagnets like Co may be assumed to be on the order of 5–15 Å. This corresponds to approximately 2–7 atomic layers. Consequently, the signal from deeper layers is exponentially suppressed by a factor of $e^{-z/\lambda}$, where λ is the IMFP.

Contribution of different sublattices at the topmost layer: Due to imperfect film growth and the presence of steps and terraces, the contribution of the A_1 and A_2 sublattices is practically unknown. This further adds to the complexity of the spectral weight distribution.

The Stoner continuum: Itinerant ferromagnets possess a broad background of single-particle excitations (the Stoner continuum) that overlaps with the collective magnon modes. This phenomenon influences the spectra in two primary ways: (i) it induces strong damping of the magnon modes through coupling to single-particle states, and (ii) it contributes an additional broad signal superimposed on the coherent magnon signals.

Mode- (layer-) dependent broadening: In reality, the magnon modes exhibit nonlinear and

mode-specific damping characteristics (59). Because the magnon wave functions in a layered structure (e.g., hcp Co films) have different spatial distributions across the atomic planes, each mode is affected differently by the local electronic environment.

The most important part of the magnon spectrum is the linear dispersion in the vicinity of the $\bar{K}$ point and the crossing at this point where the Dirac point is located. The spectra recorded here clearly show such a behavior even without any further analysis as can be seen in Figure S2(**A**). For this part we have performed two additional sets of analyses focusing on the large momentum transfer region in the vicinity of the $\bar{K}$ point. In this kinematic regime, the grazing scattering geometry significantly enhances surface sensitivity.

1. **Two-peak approach including Stoner background and variable damping:** We modeled this contribution using a linearly rising function centered at 500 meV, which corresponds to the center of the electron-pair excitation spectrum observed in both our spin-polarized and total intensity data. By fitting the spectra with variable damping rates (Γ_n) and this background, we achieve a more accurate reproduction of the difference spectra. Such an analysis is provided in Figure S2 [see the curves shown in Figure S2(**B**)].
It is worth noting that mathematically it is possible to obtain better fits by using larger peak widths. As seen in Figs. 2(**D**) and (**G**) of the manuscript and Figure S2(**B**), a close numerical agreement can be achieved by further increasing the peak widths. However, we have avoided excessive broadening to achieve these better fits, as such an approach yields damping parameters that lack a clear physical basis.

2. **Layer-resolved dynamical modeling:** To provide a rigorous physical basis for our interpretation, we implemented a comprehensive dynamical model to calculate the SPHREELS intensity $I(\omega, \boldsymbol{Q})$ based on the results of our *ab initio* calculations. The following framework forms the basis of the model used to generate the simulated results presented in Figure S2(**C**):

   (i) We construct a layer-dependent dynamical matrix using exchange coupling constants (J_{ij}) and magnetic moments (m_i) derived from first-principles. For each momentum $\boldsymbol{Q}$, we solve for the eigenvalues and eigenvectors ψ_n.

   (ii) The spin-flip scattering intensity $I(\boldsymbol{Q}, \omega)$ is modeled by projecting the layer-resolved eigenvectors $\psi_l^{(n)}(\boldsymbol{Q})$ onto a depth-attenuated basis:

$$I(\boldsymbol{Q}, \omega) \propto \sum_{n=0}^{N-1} \left| \sum_{l=1}^{N} \mathcal{A}_l(\theta) \psi_l^{(n)}(\boldsymbol{Q}) \right|^2 \frac{\Gamma_n(\boldsymbol{Q}, \omega)}{[\omega - \varepsilon_n(\boldsymbol{Q})/\hbar]^2 + \Gamma_n^2(\boldsymbol{Q}, \omega)}. \quad \text{(S4)}$$

In this framework, $\boldsymbol{Q}$ is the 2D in-plane wavevector and n is the magnon mode index ($n = 0, \dots, N-1$). The term $\psi_l^{(n)}(\boldsymbol{Q}) = \langle l|\psi_n(\boldsymbol{Q})\rangle$ represents the projection of the n-th eigenvector onto the l-th atomic layer ($l = 1, \dots, N$), while $\varepsilon_n(\boldsymbol{Q})$ denotes the magnon eigenenergy. The term $\mathcal{A}_l(\theta) = \exp(-z_l P(\theta)/2\lambda)$ accounts for the surface sensitivity of the probe, where z_l is the depth of the l-th layer and $P(\theta)$ is the geometric path-length factor which depends on the scattering angle $\theta = \theta_i$. The spectral broadening $\Gamma_n(\boldsymbol{Q}, \omega) = \eta_0 + 2\alpha|\omega|$ captures the damping resulting from the coupling of magnons to the electronic Stoner continuum. Here, η_0 represents the constant broadening and α is the dimensionless damping parameter. While we aim to employ a dynamical model targeted exclusively at the magnonic excitations, we note that it does not explicitly account for the incoherent single-particle background, which is omitted here to maintain the clarity of the peak analysis.

In Figure S2(**C**), we present simulation results along the $\bar{\Gamma}$–$\bar{K}$–$\bar{M}$ symmetry path, incorporating both surface sensitivity and damping effects as detailed above. For these simulations, we use $\eta_0 = 5$ meV and $\alpha = 0.1$. Additionally, the spectral function projected onto the topmost two atomic layers is shown in the color scale to facilitate clearer tracking of the peaks. The momentum-resolved spectral energy-loss profiles, shown by symbols in Figure S2(**C**), clearly confirm the Dirac crossing. The simulated spectra exhibit a continuous redistribution of intensity, a feature only possible if the two branches are symmetry-protected to meet at $\bar{K}$. The fact that the simulation faithfully reproduces the observed spectral features effectively rules out any significant gap-opening mechanism at this point [the symbols in Figure S2(**C**) should be compared to those shown in Figures S2(**A**) and (**B**)]. The presence of a large gap would necessitate a distinct spectral profile characterized by a bifurcated intensity distribution and a lower peak magnitude, neither of which is observed in our experimental data. Consequently, the influence of the Stoner continuum manifests as an enhancement of magnon damping and an added incoherent background, rather than as a coupling mechanism that lifts the degeneracy at the Dirac point. A critical outcome of this dynamical model is the prediction of intensity winding across the Dirac point and its observable consequences [Figure S2(**C**)]. In the multilayer geometry, the magnon eigenstates are not strictly stationary. Their relative phases and amplitudes evolve with momentum $\boldsymbol{q}$ (measured relative to the $\bar{K}$ point). An important manifestation of this winding is the redistribution of the spectral weight among the magnon branches. Specifically, the relative intensity of the α_1 modes with respect to

the α_2 modes changes notably upon crossing the Dirac point (see also Supplementary Text S7). This is clearly visible in the simulated spectra shown in Figure S2(**C**) (compare the relative intensity of the α_1 modes with respect to the α_2 modes when crossing the $\bar{K}$ point).

S3. The magnonic Bloch spectral function and magnonic density of states projected onto different layers

In order to understand the mechanism behind the formation of the magnonic bands in layered ferromagnets one way would be to calculate the atomic- (or layer-)resolved MBSF and MDOS 32(). This provides direct access to the contribution of different atoms (layers) to the magnonic band structure and allows one to unravel the origin of the observed magnon modes. A similar strategy has been used to understand the formation of the electronic bands in solids. In Figure S5 MBSF and MDOS projected onto different layers are presented. The results represent an extended version of Figure 3 of the manuscript. The MBSF projected onto all 20 layers, the layers with an odd layer index, and those with an even layer index are presented in Figures S5(**A**), (**B**) and (**C**), respectively. The corresponding total and partial MDOS are presented in Figure S5(**D**). The MBSF projected onto the top two surface layers, the topmost surface layer (the layer composed of A_1 sublattices), and the second topmost layer (the layer composed of A_2 sublattices) are presented in Figures S5(**E**), (**F**) and (**G**), respectively. The partial MDOS of the topmost and second topmost surface layers are presented in Figure S5(**H**). Likewise, the MBSF projected onto inner layers are presented in Figures S5(**I**–**K**). A careful investigation of the layer-resolved MBSF results leads to the conclusion that the magnon bands appear in pairs due to the presence of two different sublattices in the structure. This conclusion is also supported by looking at the layer-resolved MDOS, presented in Figures S5(**D**) and (**H**). The Dirac points are located in the vicinity of the crossing point of the partial MDOS, shown in Figures S5(**D**) and (**H**).

S4. Thickness dependence of the surface magnon dispersion relation

The dispersion relation of the main magnon mode observed in the experiment within the α_1 modes is shown in Figure S6. The results of the samples with different thicknesses, 3, 4, 8, 15 and 20 ML are presented. We note that for a complete hcp lattice at least three atomic layers are required. Therefore, we do not include the results of the sample with the thickness of 2 ML, in which the atoms experience a lower coordination number. The surface acoustic magnon mode of all these samples exhibits a very similar dispersion relation, except for minor differences. The results are compared to the calculated magnonic band structure and MBSF projected onto the two topmost surface layers. It is important to mention that the surface magnon mode of Co films composed of several atomic layers should be independent of the number of layers, provided the film is thick enough. In order to illustrate this, in Figure S7 we present the results of the calculations for two different thicknesses. The results are in perfect agreement with those of Michel *et al.*, which specifically focused on magnons in a small region close to the $\bar{\Gamma}$–point (50). In Figure S7(**A**) the MBSF projected onto the top two layers is presented for a 15 ML slab. The same quantity calculated for a 20 ML slab is presented in Figure S7(**B**). The results indicate that the surface modes associated with the top two layers are nearly identical, irrespective of the total number of layers. The position and the number of the bulk modes depend critically on the number of the Co layers, as expected.

One of the important results of our investigations is that the acoustic surface mode of the system is located very close or within the bulk modes. Based on a simple model and assuming the bulk values for the Heisenberg exchange parameters and the magnetic moments one expects the surface mode to be located far below the bulk modes (see Supplementary Note S6). However, unlike the localized moment magnets, in the case of itinerant magnets the pattern of both the Heisenberg exchange parameters and the magnetic moments depends strongly on the layer index (46, 66, 89). Hence, assuming the same values of exchange parameters and magnetic moments for all layers is not a valid assumption. The consequence of this fact is that the acoustic surface mode of the system is located very close to the bulk modes, instead of being located far below them. This has been manifested in the MBSF projected onto the top two layers shown in Figure S6 (see also Figures S5(**E**–**H**)).

S5. The symmetry considerations and the layer-dependent magnetic parameters

In slabs with an even number of layers, the presence of inversion symmetry ensures that the layers form pairs, effectively generating nonplanar honeycomb lattices. This symmetry enforces pairwise band crossings, which yield a Berry phase of $\pm\pi$ at the $\bar{K}$ point. In contrast, when inversion symmetry is broken, as for example in slabs with an odd number of layers, paired magnon bands can still intersect and carry a nonzero Berry phase.

Now, let us consider the case in which the slab is grown on a substrate. The presence of two different interfaces breaks the structural inversion symmetry perpendicular to the surface, even for slabs with an even number of layers. However, it is important to mention that since the magnons are the eigenstates of the spin Hamiltonian, the relevant symmetries are those of the magnetic interactions themselves. These magnetic symmetries can, however, in principle be effectively preserved or restored even when the structural inversion symmetry is broken.

Let us begin by considering the spin Hamiltonian, which includes only the symmetric Heisenberg exchange. Assuming translational invariance within each layer, one can perform an in-plane Fourier transform. This transformation allows the dynamical matrix $H(\boldsymbol{Q})$ to be expressed in momentum space as:

$$H(\boldsymbol{Q}) = \boldsymbol{M}(\boldsymbol{Q}) - \boldsymbol{N}(\boldsymbol{Q}), \tag{S5}$$

with the matrix elements:

$$M_{ll'}(\boldsymbol{Q}) = \delta_{ll'} \sum_{l''} \frac{J_{ll''}}{\sqrt{m_l m_{l''}}}, \tag{S6}$$

$$N_{ll'}(\boldsymbol{Q}) = \frac{J_{ll'}}{\sqrt{m_l m_{l'}}} e^{i\boldsymbol{Q}\cdot\boldsymbol{R}_{ll'}}. \tag{S7}$$

Here, $J_{ll'}$ represents the exchange coupling constants, while m_l and $m_{l'}$ denote the magnetic moments of the atoms in layers l and l', respectively. $\boldsymbol{R}_{ll'}$ is the real-space vector connecting magnetic layers l and l'.

The parameter that enters the matrix elements is the quantity $J_{ll'}/\sqrt{m_l m_{l'}}$. Interestingly, in most DFT-based calculations, it has been observed that at the film/substrate interface, both exchange parameters and magnetic moments decrease due to electronic hybridization. On the other hand, at the film/vacuum interface, both quantities are enhanced. This means that the matrix elements $H_{ll'}(\boldsymbol{Q})$ remain approximately symmetric when comparing the values corresponding to these two boundary layers.

In a realistic system, the details of the matrix elements depend on the atomistic magnetic parameters (e.g., interatomic exchange and atomic magnetic moments), which are themselves sensitive to structural parameters like the layer-substrate distance and substrate type. The more symmetric the matrix elements, the closer $\phi_{B,n}$ is to $\pm\pi$.

In cases where the electronic states of the substrate hybridize strongly with those of the ferromagnetic film, leading to a sizable asymmetry in the matrix elements of the dynamical matrix, one can grow a monolayer of the substrate material on top of the film in order to restore the symmetry. This architecture would guarantee the presence of inversion-symmetry-protected Dirac magnons with a Berry phase of $\pm\pi$.

S6. Magnonic band structure of hcp Co bulk vs thin films

In order to understand the role of dimensionality and the impact of itinerant electrons on the properties of the magnonic band structure, in particular the acoustic surface mode, and those of the Dirac point one may calculate the magnonic band structure of a thin slab made of a finite number of layers using the values of Heisenberg exchange parameters calculated for Co bulk. Such data are presented in Figure S8. For the sake of simplicity we only consider the interactions up to the fourth shell. The magnonic band structure of Co bulk calculated in this way is presented as the color map. The results are similar to those shown in Figure 1(**C**) of the main text. For a better comparison we also show the results of the magnonic band structure of Co bulk when all the interactions are considered (open symbols, the data are the same as those shown in Figure 1(**C**)). The minor differences between these two results are due to the limited number of shells considered in the present calculations. The differences manifest the importance of the Heisenberg exchange parameters describing the interaction between neighbors located at larger distances and the role of the itinerant electrons in these interactions, being mainly of RKKY type ($90-92$). The solid and dashed curves in Figure S8(**A**) represent the magnonic band structure calculated for a slab of hcp Co using the same values of the Heisenberg exchange parameters and magnetic moments. The results clearly show two sets of modes: (i) bulk modes represented by the white solid curves and (ii) two degenerate acoustic surface modes located below the bulk modes, represented by the dashed red and green curves. The calculations performed for a 15 ML slab are shown in Fig S8(**B**), confirming that the acoustic surface modes of the two systems are identical. This observation that the acoustic surface modes are located far below the bulk modes is due to the fact that the appearance of these modes in the present examples is solely due to the fact that the coordination number of Co atoms sitting in the two surface layers (on the top and bottom of the slab) is lower than the atoms sitting in the inner part of the film. This leads to the formation of two surface modes located below the bulk modes, which degenerate over a large fraction of the surface BZ. It is important to emphasize that in real systems due to the itinerant nature of magnetism of Co both the Heisenberg exchange parameters as well as the magnetic moments for atoms sitting in different layers are no longer the same. In particular, it is often observed that both of these quantities are enhanced near the surface. All these points have been taken into account in the calculations shown throughout the manuscript (see also Methods). The enhanced Heisenberg exchange parameters and magnetic moments at the surface leads to fact is that the acoustic surface mode of the Co films to be located in the close vicinity or within the bulk modes, instead of being located far below them. Moreover, the presence of the substrate affects both the Heisenberg exchange parameters and magnetic moments in the layers close to the interface. Hence, the pattern of the Heisenberg exchange parameters

and magnetic moments can be very complex in real systems. This leads to differences between the magnonic band structure of bulk and real thin films grown on a substrate. In particular if the film is only a few atomic layers thick, the differences would be substantial $(42, 46, 54, 93-95)$. For rather thick films, like the samples discussed here, one can safely exclude the interfacial effects, when mainly the topmost layers are probed. Owing to the surface sensitivity of SPHREELS, this is a valid and fully justified assumption $(46, 49, 80)$.

S7. Intensity winding around the $\bar{K}$–point

S7.1. Origin of the intensity modulation around the Dirac point

One consequence of a Berry phase of magnitude π (mod 2π) acquired by encircling a Dirac point is a modulation of the magnon intensity in momentum space around that point, as shown in Refs. (29, 30). To determine the extent to which a similar intensity modulation is expected in SPHREELS spectra, we begin by considering the bulk case.

Consider a minimal two-sublattice (A_1/A_2) tight-binding (or linear-spin-wave) hopping amplitude

$$\gamma(\boldsymbol{Q}) = t \sum_{j=1}^{3} e^{i\boldsymbol{Q}\cdot\boldsymbol{\delta}_j},$$

where $\boldsymbol{Q}$ is the magnon momentum and $\boldsymbol{\delta}_j$ ($j = 1, 2, 3$) are the three nearest-neighbor vectors of the honeycomb network. At the high-symmetry K point the sum vanishes so that $\gamma(\boldsymbol{K}) = 0$. Expanding about the K point, $\boldsymbol{Q} = \boldsymbol{K} + \boldsymbol{q}$ with $|\boldsymbol{q}| \ll |K| \sim 1/a$, gives

$$\gamma(\boldsymbol{K} + \boldsymbol{q}) \simeq v\,(q_x + iq_y) \equiv v\,q\,e^{i\varphi},$$

where $q = |\boldsymbol{q}|$, $\varphi = \arg(q_x + iq_y)$, and v is a velocity set by t and the lattice geometry. This linear dispersion is the Dirac (massless) approximation to the bands near K.

Neglecting diagonal terms, the minimal two-band Bloch Hamiltonian is

$$\mathcal{H}(\boldsymbol{q}) = \begin{pmatrix} 0 & \gamma(\boldsymbol{q}) \\ \gamma^*(\boldsymbol{q}) & 0 \end{pmatrix},$$

with $\gamma(\boldsymbol{q}) = vqe^{i\varphi}$ and eigenvalues $\hbar\omega_\pm = \pm vq$ (measured relative to the Dirac-point frequency). Choosing the gauge that makes the first spinor component real, the normalized eigenvectors are

$$\psi_\pm(\boldsymbol{q}) = \frac{1}{\sqrt{2}} \begin{pmatrix} 1 \\ \pm e^{i\phi(\boldsymbol{q})} \end{pmatrix}, \qquad \phi(\boldsymbol{q}) = \arg\gamma(\boldsymbol{q}) \simeq \varphi.$$

Thus the relative phase between A_1 and A_2 components is $\phi(\boldsymbol{q})$. Upon encircling the Dirac point, $\phi(\boldsymbol{q})$ winds by 2π. The Berry connection for this two-component spinor evaluates to

$$\boldsymbol{A}_\pm(\boldsymbol{q}) \equiv i\langle\psi_\pm|\nabla_{\boldsymbol{q}}\psi_\pm\rangle = -\tfrac{1}{2}\nabla_{\boldsymbol{q}}\phi(\boldsymbol{q}),$$

so that a 2π winding of ϕ yields a Berry phase ϕ_B (up to sign/convention)

$$\phi_B = \oint \boldsymbol{A}_\pm \cdot d\boldsymbol{q} = -\tfrac{1}{2}\oint d\phi = \pm\pi,$$

whose magnitude is π for a single encirclement (the overall sign depends on orientation and gauge).

S7.2. Neutron scattering intensity near the Dirac point

The coherent magnetic structure-factor amplitude for a mode (relevant to neutron scattering) is the sum of sublattice contributions projected onto the scattering sensitivity:

$$\mathcal{F}_{\pm}(\boldsymbol{q}) \propto F_{A_1} u_{A_1} + F_{A_2} u_{A_2} e^{i\boldsymbol{q}\cdot\boldsymbol{\tau}},$$

where $u_{A_{1,2}}$ are eigenvector components, $F_{A_{1,2}}$ are form factors, and $\boldsymbol{\tau}$ is the A_1–A_2 basis offset. For equal form factors ($F_{A_1} = F_{A_2}$) and using $u_{A_1} = 1/\sqrt{2}$, $u_{A_2} = \pm e^{i\phi(\boldsymbol{q})}/\sqrt{2}$, we obtain

$$\mathcal{F}_{\pm}(\boldsymbol{q}) \propto \frac{1}{\sqrt{2}}\big[1 \pm e^{i(\phi(\boldsymbol{q})+\phi_0)}\big], \qquad \phi_0 \equiv \boldsymbol{q}\cdot\boldsymbol{\tau}.$$

Hence the measured intensity (up to a global prefactor and polarization factors) is

$$I_{\pm}(\boldsymbol{q}) \propto \big|1 \pm e^{i(\phi(\boldsymbol{q})+\phi_0)}\big|^2 = 2\big[1 \pm \cos(\phi(\boldsymbol{q}) + \phi_0)\big].$$

Using $\phi(\boldsymbol{q}) \simeq \varphi$ in the linear approximation yields the sinusoidal angular modulation observed experimentally in Refs. (29,30):

$$I_{\pm}(\varphi) \propto 2\big[1 \pm \cos(\varphi + \phi_0)\big].$$

Here the $\pm$ sign corresponds to the two Dirac branches, or equivalently to symmetric/antisymmetric A_1–A_2 combinations. The sign flip between the $+$ and $-$ branches explains the inversion of intensity above vs. below the Dirac crossing. The phase $\phi_0 = \boldsymbol{q}\cdot\boldsymbol{\tau}$ arises from the relative displacement of the sublattices. While the individual phases $\phi(\boldsymbol{q})$ and ϕ_0 depend on the choice of unit-cell origin, their sum, and thus the physical intensity $I\pm(\boldsymbol{q})$, is gauge-invariant and uniquely determined by the crystal symmetry and scattering geometry. Unequal form factors ($F_{A_1} \neq F_{A_2}$) deform the amplitude but do not remove the leading winding unless they induce a sizable gap or strongly suppress one sublattice contribution. Such scenarios are unlikely in typical elemental, single-crystalline bulk samples.

S7.3. Finite slab of N hcp(0001) layers probed with a surface-sensitive method

Let us now consider a slab of N atomic layers stacked along the crystallographic c-axis (the hcp(0001) direction). The two important and distinct differences from the bulk are:

1. *Broken translational symmetry along* Q_z: the bulk continuous Q_z is quantized into N discrete standing-wave modes. For open boundary conditions the approximate quantization is $Q_{z,n} \approx 2n\pi/Nc$ with $n = 0, \ldots, N-1$. The factor of 2 arises because the hcp unit cell contains two layers per c. The bulk nodal line that extended continuously along Q_z is therefore sampled at discrete $Q_{z,n}$, giving a set of 2D Dirac crossings for each n. For $N = 20$ one expects 20 quasi-2D subbands that inherit the Dirac character near the in-plane $\bar{K}$–points.

2. *Surface-localized modes and modified eigenvectors*: surfaces break the A_1/A_2 balance and modify local exchange. The eigenstates split into bulk-like (extended) and surface-localized components.

Considering only the surface sensitivity: It is important to notice that the physical interaction between the scattering particles and the sample responsible for the excitation of magnetic excitations is a very important factor in determining the spectral intensity. Focusing on the magnetic component of the scattering, neutrons are charge-neutral particles that couple to the magnetic dipoles within a sample. This interaction is effectively described by the Fourier transform of the spin density. The dynamical structure factor is the physical quantity that sets neutron intensity. In contrast, electrons carry a negative charge and hence low-energy electrons scatter primarily through an *exchange* interaction which is of Coulomb nature, involving the spin density or exchange field of the solid (see Ref. (80) and references therein for an extended discussion). In particular in SPHREELS the spin-flip (transverse) part of the electronic scattering amplitude is what excites magnons. The scattering matrix elements are therefore closer to a localized exchange overlap between the incident electron wavefunction and that of the surface electrons. For the sake of simplicity let us now consider that the electrons behave exactly like neutrons and are only more surface sensitive. We aim to understand how the finite number of layers within the slab geometry and the surface sensitivity would affect the intensity winding under such an assumption.

Starting from the coherent amplitude idea used for bulk hcp lattice with A_1 and A_2 sublattices and introducing a surface weight $w(z)$ and possibly layer-dependent eigenvector components $u_{A_1,A_2}(z)$ and a geometry phase $\phi_0^{(\mathrm{el})}$, one may write the structural factor in the form:

$$\mathcal{F}(\boldsymbol{q}) \propto \sum_{z=1}^{N} w(z)\big[\, u_{A_1}(z) + u_{A_2}(z)\, e^{i(\phi_0^{(\mathrm{el})}+\phi(\boldsymbol{q}))}\,\big].$$

The measured intensity would therefore be:

$$I(\varphi) = \big|\mathcal{F}(\boldsymbol{q})\big|^2 = \Big|\sum_z w(z)\big[u_{A_1}(z) + u_{A_2}(z) e^{i(\phi_0^{(\mathrm{el})}+\phi(\boldsymbol{q}))}\big]\Big|^2,$$

where $\phi(\boldsymbol{q})$ is the Bloch eigenvector phase (the Dirac winding $\sim \varphi$) and $\phi_0^{(\mathrm{el})}$ is the electron-scattering offset phase (includes basis offset and electron path phases). For a strongly surface-sensitive probe, $w(z)$ is sharply peaked at small z, so the sum is dominated by the top layer(s).

Given the above assumptions, one can consider the two following scenarios:

- If the surface preserves the A_1–A_2 balance and $u_{A_1}(z=1) = u_{A_2}(z=1)$ (as in bulk-like eigenvectors), and w picks the top layer, one recovers the familiar interference:

$$I_{\pm}(\varphi) \propto |1 \pm e^{i(\phi(\boldsymbol{q})+\phi_0^{(\mathrm{el})})}|^2 \sim 2[1 \pm \cos(\varphi + \phi_0^{(\mathrm{el})})].$$

One would therefore expect the same sinusoidal winding and the upper/lower inversion but now with enhanced contrast, because the interference comes predominantly from the top layers where the phase is coherent. We note that a perfect slab with a fixed number of layers cannot preserve the A_1–A_2 balance, since this would be incompatible with the symmetry of the hcp(0001) slab. Only if the electrons "see" all the slab uniformly would one end up with the bulk scenario.

- If the surface breaks sublattice symmetry (e.g., termination on A_1 sites or modified surface exchange so $u_{A_1}(z=1) \neq u_{A_2}(z=1)$), write $\mathcal{A} \equiv \sum_z w(z) u_{A_1}(z)$, $\mathcal{B} \equiv \sum_z w(z) u_{A_2}(z)$. Then

$$I_\pm(\varphi) \propto |\mathcal{A} \pm \mathcal{B} e^{i(\phi+\phi_0^{(\mathrm{el})})}|^2 = \mathcal{A}^2 + \mathcal{B}^2 \pm 2\mathcal{A}\mathcal{B}\cos(\phi + \phi_0^{(\mathrm{el})}) \tag{S8}$$

  The modulation depth scales as $2\mathcal{A}\mathcal{B}/(\mathcal{A}^2 + \mathcal{B}^2)$. If one sublattice is strongly suppressed at the surface ($\mathcal{A} \ll \mathcal{B}$ or vice versa), the modulation collapses (vanishes in the extreme case) because there is no two-path interference.

From the two limiting cases discussed above, one would conclude (i) there should be an intensity winding around the $\bar{K}$–point, and (ii) the intensity modulation is expected to be considerably reduced compared to the bulk case.

As noted earlier, the scattering mechanism responsible for magnon creation differs when electrons are used as scattering probes. Consequently, the following aspects merit consideration:

Momentum and phase sensitivity: The electron scattering amplitude includes phase factors arising from the electron plane wave (or scattering state) at each atomic site. The total relative phase $\phi_0^{(\mathrm{el})}$ between A_1 and A_2 contributions at the surface therefore includes contributions from the surface geometry, the incidence and detection angles of the electrons, and the local positions of the basis atoms. It is not determined solely by the bulk basis vector. The electron momentum perpendicular to the surface is not a conserved quantity. These effects enter as $\phi_0^{(\mathrm{el})}$, which can be large and dependent on the experimental geometry.

Stronger coupling and larger backgrounds: Electron–magnon matrix elements can be significantly larger compared to those for neutrons, resulting in a stronger scattering cross-section per surface atom. However, a major challenge in inelastic electron spectroscopy is the presence of a larger electronic background and the effect of multiple scattering. These factors lead to line broadening and can obscure or mask weak interference features.

Penetration depth and weighting: Typical inelastic mean free paths for low-energy electrons are a few angstroms up to a few nanometers, depending on the incident energy. This means that the measured response is a coherent superposition dominated by the top few layers, effectively a weight profile $w(z)$ that decays rather quickly into the inner part of the slab. The layer-dependent weight

$w(z)$ is not known exactly from experiment, but it typically decays roughly exponentially with depth, giving most of the signal (≈80–95%) to the top few atomic layers. This profile effectively limits the coherent interference to the surface layers in strongly surface-sensitive measurements.

Considering all the aspects discussed above, one expects the intensity modulation in SPHREELS experiments on thin films to be considerably weaker than in bulk measurements. In addition, spectral broadening from Landau damping and the coexistence of multiple magnon branches make it difficult to perform reliable intensity analysis at single energy points or exactly at the Dirac crossings. Consequently, such analyses should be carried out by integrating the intensity over a finite energy window and at in-plane momenta slightly away from the $\bar{K}$ point.

Probing the intensity map around the $\bar{K}$ is highly challenging because it requires the simultaneous rotation of the sample around two axes: one perpendicular to the surface and one parallel to the surface. Moreover, the polarization vector of the incident beam should also be rotated in the laboratory framework so that it is always parallel to the quantization axis (the direction of the sample magnetization). The realization of such an experimental setup is highly demanding. However, it is possible to probe the magnons on the two sides of the $\bar{K}$–point, i.e., at $\bar{K} \pm q$. Examples of the spectra recorded at $\bar{K} \pm 0.35$ Å^{-1} are shown in Figure S9(**A**), where the difference spectra recorded at $\bar{K} \pm q$ with $q = 0.35$ Å^{-1} are presented. The spectra were recorded at incident energies of $E_i = 5.0$ and 19.0 eV for $\bar{K} - 0.35$ and $\bar{K} + 0.35$ Å^{-1}, respectively. The data clearly indicate that the relative intensity of α_1 with respect to that of α_2 depend critically on the sign of q. Since the absolute values of the intensities on the two sides of the $\bar{K}$–point depend on several factors including scattering angles, incident electron energy, and pass energies, it is very useful to analyze the ratio of the intensities over an energy window close to the α_1 and α_2 modes. The analysis of the intensity maps based on these new data are provided in Figure S9(**B**) for $\bar{K} \pm q$, with q between 0.25 and 0.35 Å^{-1}, $\hbar\omega_1 = 325 \pm 30$ meV (below the Dirac point) and $\hbar\omega_2 = 390 \pm 20$ meV (above the Dirac point), showing the expected intensity winding. Here the $\boldsymbol{q}$-resolved intensity ratio $\mathcal{R}(\text{II}) = I_{\hbar\omega_1=325\pm30}/I_{\hbar\omega_2=390\pm20}$ is presented. In addition, $\mathcal{R}(\phi(\boldsymbol{q}))$ as a function of $\phi(\boldsymbol{q})$ for several values of q between 0.25 and 0.35 Å^{-1} is shown in Figure S9(**C**). The primary conclusion of these findings is that the nonzero Berry phase associated with the magnon excitations manifests as a characteristic intensity winding in the spectra, which is clearly observable in the experimental data.

S.8. Comprehensive evidence for Dirac magnon formation in hcp Co films

While broad spectral features are an inherent physical property of itinerant magnets, the identification of a 2D Dirac crossing is in hcp Co films is supported by a self-consistent framework comprising several independent experimental pillars:

(i) **Dispersion dynamics and degenerate crossings.**

(ii) **Spectral weight evolution.**

(iii) **Topological signatures and intensity winding.**

In the following we discuss them in details.

S8.1. Dispersion dynamics and degenerate crossings

When tracking the modes along the $\bar{\Gamma}$–$\bar{K}$–$\bar{M}$ symmetry path, one observes a clear closing and reopening of the gap. This was illustrated in Figures S2(**A**) and S2(**B**), where a series of difference spectra recorded in the immediate vicinity of the $\bar{K}$ point is shown. The data and the corresponding fits confirm that as the momentum approaches the $\bar{K}$ point, the two distinct peaks (α_1 and α_2) merge. This mode evolution serves as an experimental signature of a degenerate crossing, persisting despite the considerable intrinsic broadening of the individual modes.

S8.2. Spectral weight evolution

Along the $\bar{\Gamma}$–$\bar{K}$–$\bar{M}$ symmetry path, the two distinct magnon branches, α_1 and α_2, exhibit a clear convergence. As shown in Figure S2(**A**), SPHREELS difference spectra recorded in the immediate vicinity of the $\bar{K}$ point confirm that as momentum approaches the high-symmetry point, the two modes merge into a single, intensified feature. The analytical fits (Figure S2(**B**)), based on the Voigt-profile methodology described in Supplementary Note S2, capture this redistribution of spectral weight. The convergence results in a narrower peak with enhanced magnitude, serving as a characteristic experimental signature of a degenerate crossing. This transition from a double-peak structure to a single intensified resonance provides direct evidence against any gap-opening mechanisms or substantial degeneracy lifting induced by the Stoner continuum.

Constant-energy manifolds: Analysis of constant-energy maps at the predicted Dirac energy (shown in Figure 4 of the manuscript) also reveals a concentration of spectral weight at the $\bar{K}$ point, indicating the coalescence of the magnon branches.

Layer-resolved dynamical modeling: As shown in Figure S2(**C**), the simulated profiles indicate a continuous redistribution of intensity that is only possible if the two branches are symmetry-protected to meet at $\bar{K}$. The absence of a bifurcated intensity distribution in both the simulation and the experiment rules out significant gap-opening mechanisms.

S8.3. Topological signatures and intensity winding

The most critical evidence for the topological nature of the crossing is the intensity winding around the $\bar{K}$ point. As discussed in Supplementary Note S7, a Dirac point characterized by a $\pm\pi$ Berry phase dictates a specific modulation of the magnon intensity. In the hcp lattice, the intensity $I_{\pm}(\varphi)$ follows a sinusoidal behavior. As shown in Figure S2(**C**), the data at $\bar{K} \pm q$ clearly exhibit a distinctive signature: the relative intensity ratio of the α_1 and α_2 modes reverses upon crossing the Dirac point. This intensity winding is a topological property originating from the phase texture of the magnonic wavefunctions. Its observation provides compelling evidence of a symmetry-protected Dirac node, a feature that is also clearly visible in Figure S9.

□ Supplementary Table

Table S1.

Table S1. **High-symmetry points and the expected Berry phase for ideal layered systems of hcp surfaces.** Only symmetric Heisenberg exchange has been taken into account. The main conclusion of these results is that a Berry phase of $\pm\pi$ is only expected for multilayer films of (0001) type (thicknesses above one atomic layer).

**Structure**	**Surface BZ shape**	**High-symmetry points**	**Expected Berry phase**
hcp bulk	hexagonal	Γ, A, H, K, M, L	0, 0, 0, $\pm\pi$, 0, 0
hcp(0001)	hexagonal	$\bar{\Gamma}$, $\bar{\mathrm{K}}$, $\bar{\mathrm{M}}$	0, $\pm\pi$ (multilayers), 0
hcp($10\bar{1}0$)	rectangular	$\bar{\Gamma}$, $\bar{\mathrm{X}}$, $\bar{\mathrm{Y}}$, $\bar{\mathrm{M}}$	0, 0, 0, 0
hcp($11\bar{2}0$)	rectangular	$\bar{\Gamma}$, $\bar{\mathrm{X}}$, $\bar{\mathrm{Y}}$, $\bar{\mathrm{M}}$	0, 0, 0, 0

□ Supplementary Figures

Figure S1.

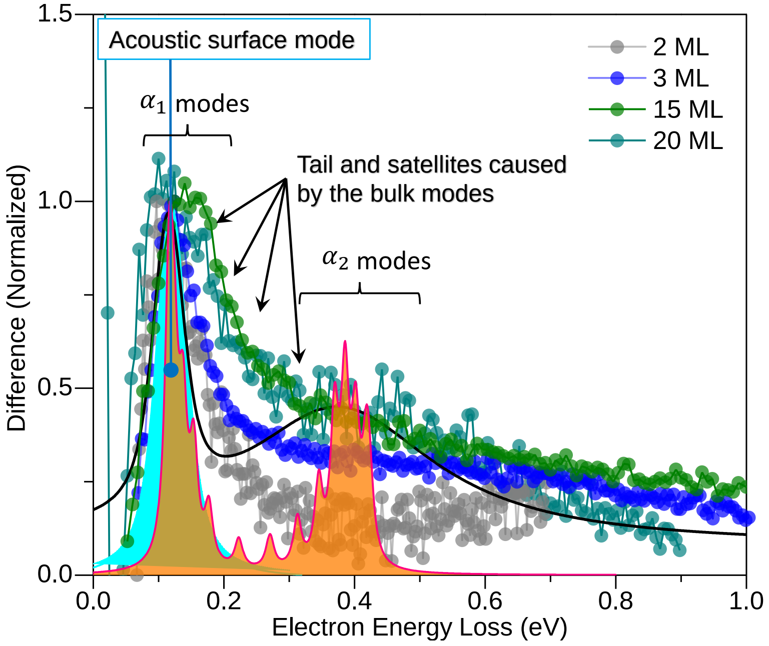

Figure S1. **Thickness dependence of the SPHREELS spectra.** The difference spectra recorded at $|\boldsymbol{Q}| = 0.6$ Å^{-1} on Co films of different thicknesses. For the sake of better comparison each spectrum is normalized to its maximum intensity. In the case of 2 and 3 ML Co the spectra are dominated by the $n = 0$ acoustic surface mode, which appears as a narrow and well-defined peak. The higher order modes with $n \neq 0$ appear at higher energies and are, therefore, well separated from the $n = 0$ mode. For example, in the case of the 3 ML sample the $n = 1$ mode is located at an energy of about 200 meV. In contrast, for the samples with the thickness of 15 and 20 ML the $n \neq 0$ modes are densely packed and appear as a tail and satellites above the acoustic surface mode. The calculated spectral function for a 15 ML sample is shown by the red solid curve with the orange shaded area for a comparison. The black curve is a function including two peak functions representing the α_1 and α_2 modes, each of which can in turn include several magnon modes. The main contributions to these modes are the acoustic and optical surface modes, respectively.

Figure S2.

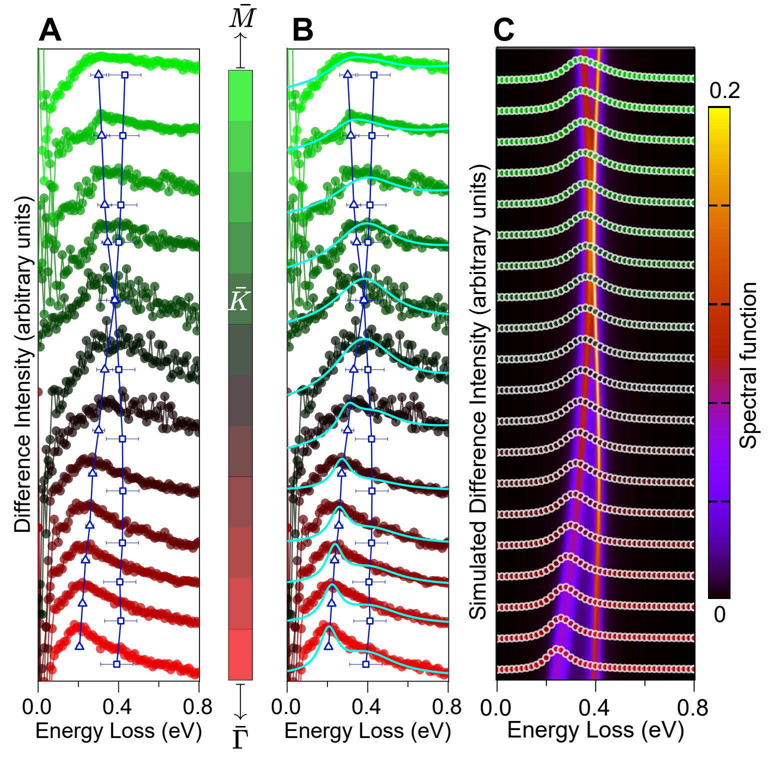

Figure S2. **Linear magnon dispersion relation and Dirac crossing at the $\bar{K}$–point.** (**A**) Series of normalized difference spectra recorded along the $\bar{\Gamma}$–$\bar{K}$–$\bar{M}$ symmetry path across the $\bar{K}$–point, indicating a linear dispersion in the vicinity of the high-symmetry $\bar{K}$–point and a Dirac crossing at this point. The data represent a magnified version of Figure 2(**I**). Spectra are scaled for clarity. The experimental data show the α_1 and α_2 modes converging toward a singular crossing at the high-symmetry point. (**B**) Corresponding Voigt-profile fits based on the methodology described in Supplementary Note S2. (**C**) Simulated momentum-resolved spectral energy-loss profiles along the $\bar{\Gamma}$–$\bar{K}$–$\bar{M}$ symmetry path, incorporating both surface sensitivity and damping effects. For this simulations we use $\eta_0 = 5$ meV and $\alpha = 0.1$. Additionally, the spectral function projected onto the topmost two atomic layers is shown to facilitate a clearer tracking of the peaks.

Figure S3.

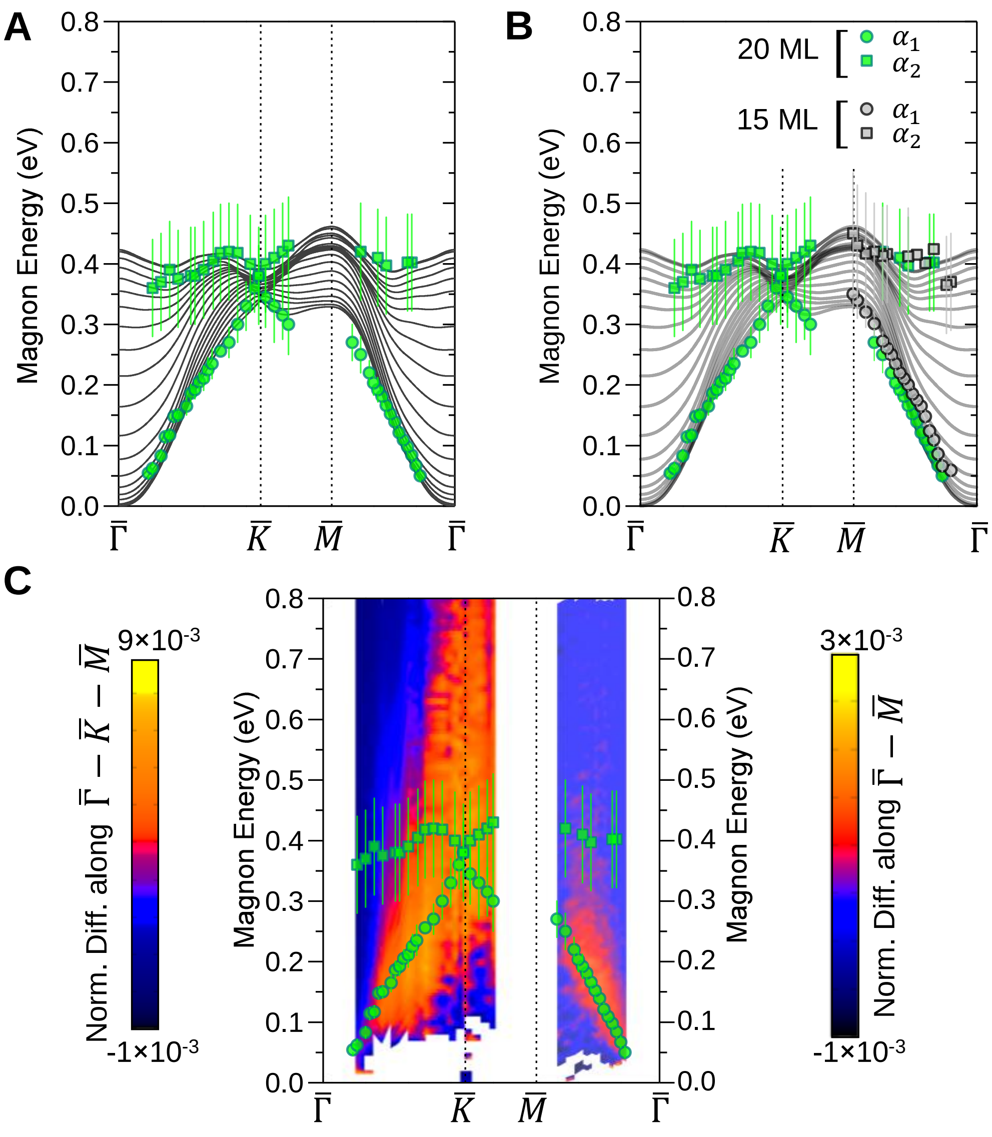

Figure S3. **An extended version of Figure 3: The magnon dispersion relation of thin hcp Co films on W(110).** (**A**) The magnonic band structure calculated based on first principles. The experimental magnon energies of the α_1 and α_2 modes shown by the solid symbols. (**B**) A comparison between the results of the 20 ML sample and that of the 15 ML one. The experimental results of the 15 ML sample are recorded along the $\bar{\Gamma}$–$\bar{M}$ direction only. (**C**) The intensity map of a 20 ML Co film, as presented in Figure 3 of the main manuscript.

Figure S4.

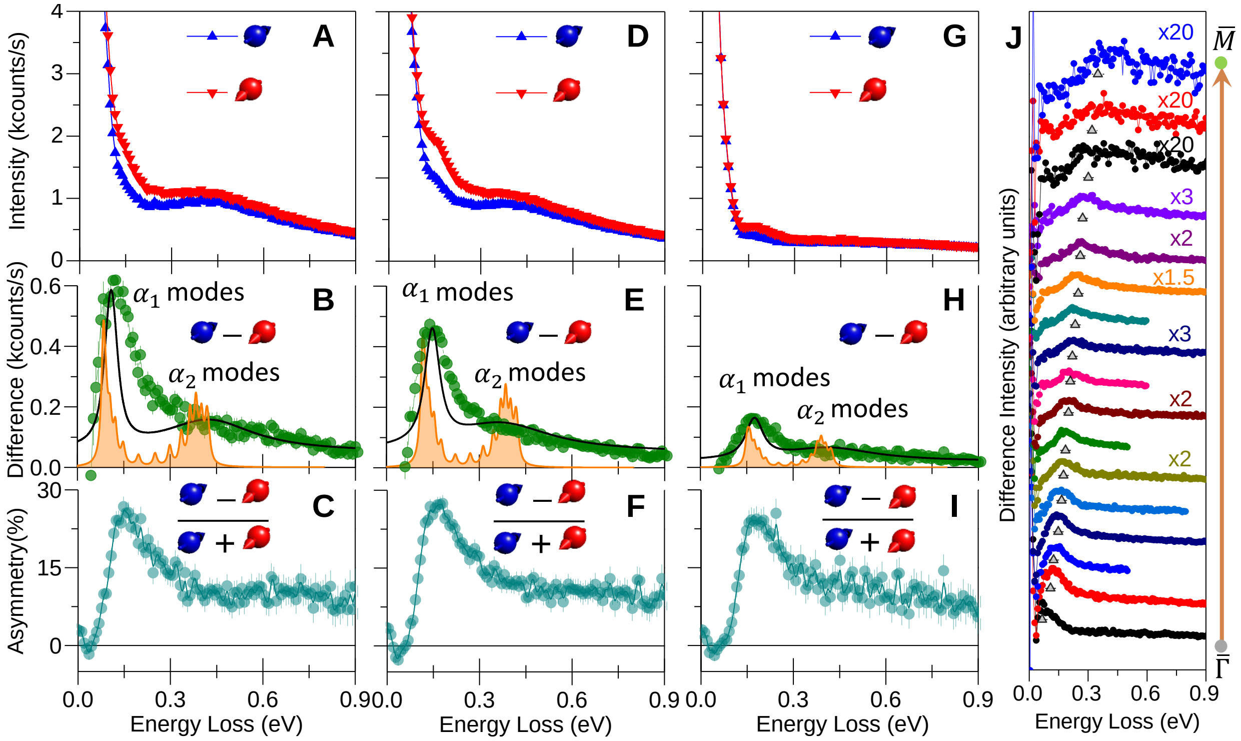

Figure S4. **SPHREELS spectra recorded on a 15 ML thick film of hcp Co on W(110).** (**A**–**I**) Typical SPHREELS spectra recorded on 15 ML Co on W(110) at the incident energy of $E_i = 4$ eV. The spectra were recorded at the wavevector transfers of $|\boldsymbol{Q}| = 0.5$ Å^{-1} (**A**–**C**), $|\boldsymbol{Q}| = 0.6$ Å^{-1} (**D**–**F**) and $|\boldsymbol{Q}| = 0.7$ Å^{-1} (**G**–**I**). The spin-polarized spectra are shown in (**A**), (**D**) and (**G**), the difference spectra are shown in (**B**), (**E**) and (**H**) and the asymmetry spectra are presented in (**C**), (**F**) and (**I**). The spectra shown in red and blue were recorded with the spin polarization vector of the incident electron beam being parallel and antiparallel to the scattering plane's normal vector $\hat{n}$, respectively. In (**B**), (**e**) and (**G**) the spectral function calculated based on first principles is also shown by the orange color for a comparison. One recognizes the presence of two classes of magnon modes, denoted by α_1 and α_2. (**J**) Series of difference spectra recorded along the $\bar{\Gamma}$–$\bar{M}$ high-symmetry direction. The spectra below (above) $|\boldsymbol{Q}| = 1.2$ Å^{-1} were recorded at $E_i = 4$ eV ($E_i = 7$ eV). For the sake of clarity some spectra are scaled as indicated. The energy dispersion of the α_1 modes is shown by the triangles. All the spectra were recorded at room temperature.

Figure S5.

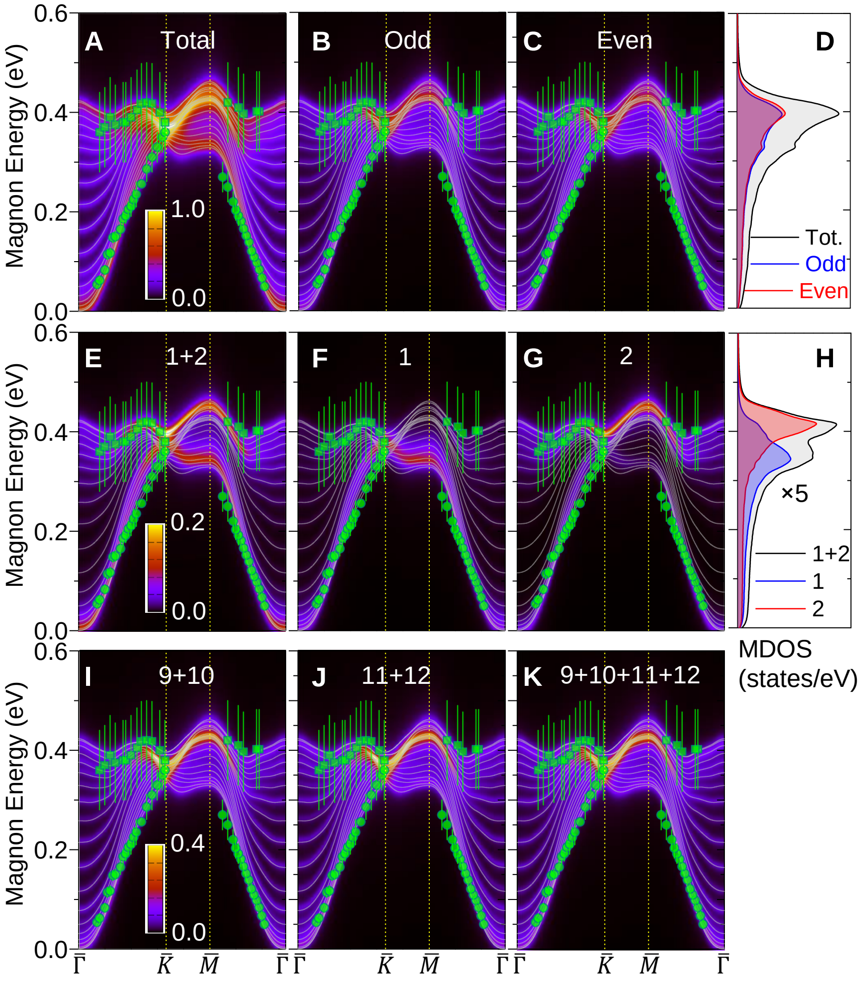

Figure S5. **Magnonic Bloch spectral function (MBSF) and magnonic density of states (MDOS) of a 20 ML hcp Co film.** (**A**) MBSF of all 20 layers. (**B**) MBSF projected onto the layers with an odd layer index. (**C**) MBSF projected onto the layers with an even layer index. (**D**) MDOS projected onto the layers with odd and even layer indices. The total MDOS is shown as well. (**E**) MBSF projected onto the top two surface layers (layer indices 1 and 2). (**F**) MBSF projected onto the topmost surface layer (layer index 1). (**G**) MBSF projected onto the second topmost surface layer (layer index 2). (**H**) MDOS projected onto the top two surface layers. (**I**) MBSF projected onto the layers with the indices 9 and 10, (**J**) MBSF projected onto layers with the indices 11 and 12, (**K**) MBSF projected onto layers with the indices 9 to 12. The magnonic band structure is also shown by solid lines in (**A**–**C**), (**E**–**D**), and (**I**–**K**).

Figure S6.

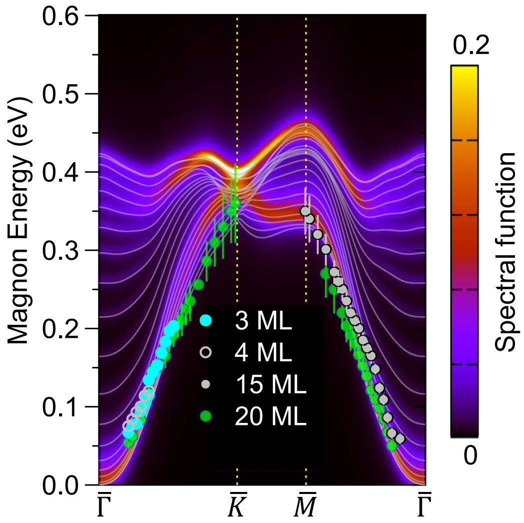

Figure S6. **Dispersion relation of the acoustic surface magnon mode of hcp Co films of different thicknesses grown on W(110).** The experimental data are shown by the symbols and the calculated magnonic band structure for a 20 ML film is shown by the solid curves. The color map represents the MBSF of a 20 ML hcp Co slab projected onto the top two surface layers.

Figure S7.

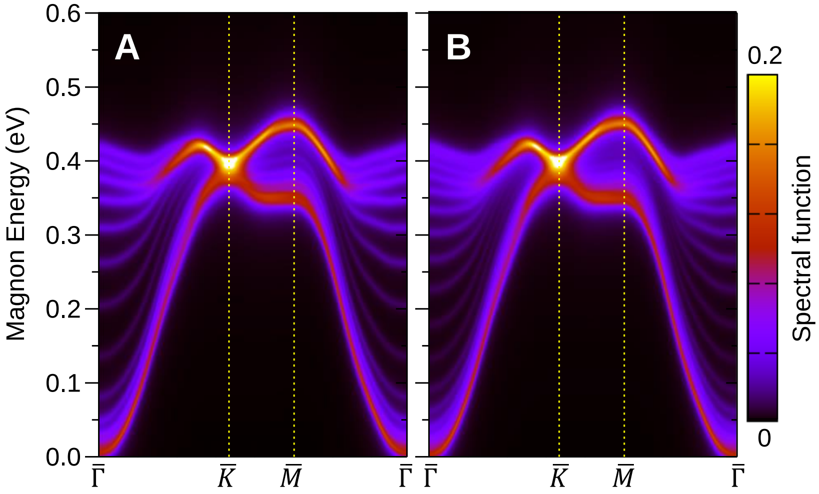

Figure S7. **Thickness dependence of the magnonic Bloch spectral function of the two topmost surface layers.** The results of the sample with a thickness of 15 ML are presented in (**A**) and those of the 20 ML sample are presented in (**B**). The data demonstrate that in contrast to the bulk modes both the acoustic and optical surface modes of the two system are nearly identical.

Figure S8.

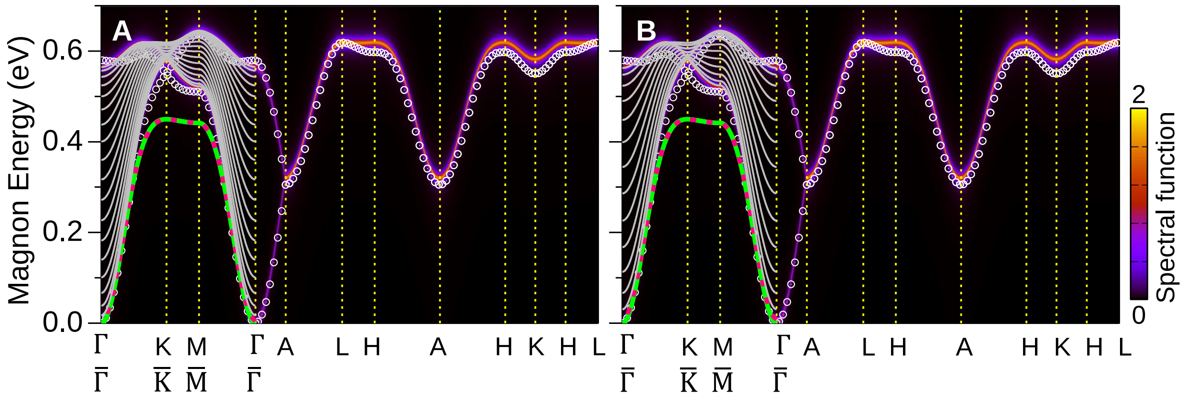

Figure S8. **A comparison between the magnonic band structure of hcp Co bulk and thin films.** (**A**) MBSF calculated for Co bulk considering the Heisenberg exchange parameters only up to the fourth shell. The solid and dashed curves represent the magnonic band structure of a 20 ML slab of Co using the bulk parameters. Here again the interactions up to the fourth shell are considered. The dashed green and red curves represent the acoustic surface modes located far below the bulk modes (white solid curves). The open symbols represent the bulk calculations when all the Heisenberg exchange parameters are considered. The data are the same as those presented in Figure 1(**C**) of the manuscript. (**B**) The same as (**A**) but for a 15 ML slab. Again the dashed green and red curves represent the acoustic surface modes of the system which are identical to those of the 20 ML slab shown in (**A**).

Figure S9.

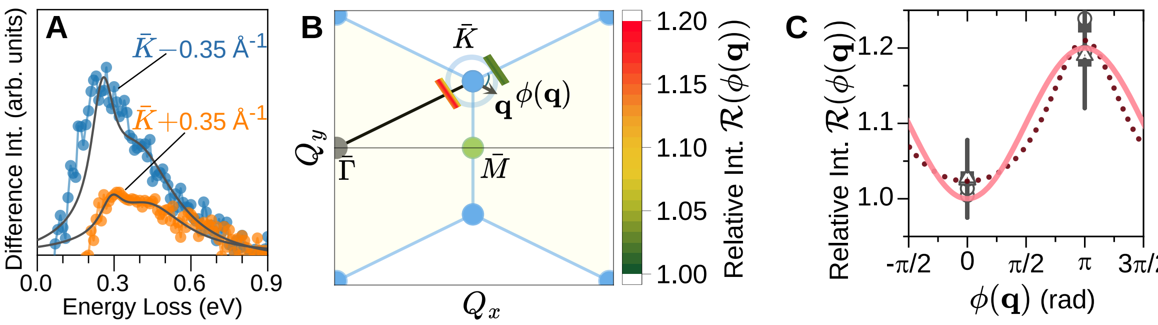

Figure S9. **Intensity winding around the $\bar{K}$–point.** (**A**) Difference spectra recorded at $\bar{K} \pm q$ with $q = 0.35$ Å^{-1}. The spectra were recorded at and incident energy of $E_i = 5.0$ and 19.0 eV for $\bar{K} - 0.35$ and $\bar{K} + 0.35$ Å^{-1}, respectively. Hence, the absolute value of the intensities cannot be directly compared. The data clearly indicate that the relative intensity of α_1 and α_2 modes depends critically on the sign of q. (**B**) The map of the relative intensity of $\hbar\omega_1 = 325 \pm 30$ meV (below the Dirac point) and $\hbar\omega_2 = 390 \pm 20$ meV (above the Dirac point) in the momentum space. $\mathcal{R}(\boldsymbol{q}) = I_{\hbar\omega_1 = 325\pm30}/I_{\hbar\omega_2=390\pm20}$ is shown for $\bar{K} \pm q$, with $0.25 < q < 0.35$ Å^{-1}, showing the expected intensity winding. The circle around the $\bar{K}$ marks the momentum region, where the intensity map is recorded. (**C**) $\mathcal{R}$ collected around the $\bar{K}$–point with q between 0.25 and 0.35 Å^{-1} as a function of the $\phi(\boldsymbol{q})$. Solid and dotted curves show the profile expected based on Eq. (S8), solid curve: $\mathcal{R} = 0.1[1 - \cos(\phi(\boldsymbol{q}))] + 1$, dotted curve: $\mathcal{R} = 0.07\{[1 - \cos(\phi(\boldsymbol{q}))] + 1\}/\{[1 + \cos(\phi(\boldsymbol{q}))] + 1\}$. The data points in (**C**) represent the result of five pairs of data points recorded at different values of the in-plane wave vector $\boldsymbol{q}$.

Figure S10.

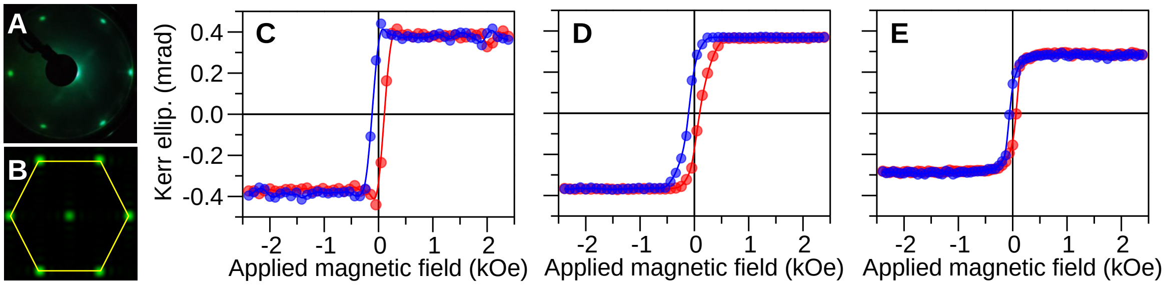

Figure S10. **Structural and magnetic characterizations of hcp Co films on W(110).** (**A**) A LEED pattern recorded at an incident electron energy of 80.2 eV on a sample with the thickness of 20 ML. (**B**) The corresponding simulated LEED pattern, likewise for an incident electron energy of 80.2 eV. (**C**) The MOKE hysteresis loop recorded on the 20 ML sample, when the magnetic field was applied along the Co[$1\bar{1}00$] direction (or W[$1\bar{1}0$] direction). (**D**) The same as (**C**) but with the magnetic field applied along the Co[$11\bar{2}0$] direction (or W[100] direction). (**E**) The hysteresis loop of a 15 ML sample with the magnetic field applied along the Co[$11\bar{2}0$] direction (or W[100] direction). The blue and red colors represent the downward and upward field scan, respectively. All the data were recorded at room temperature.

Figure S11.

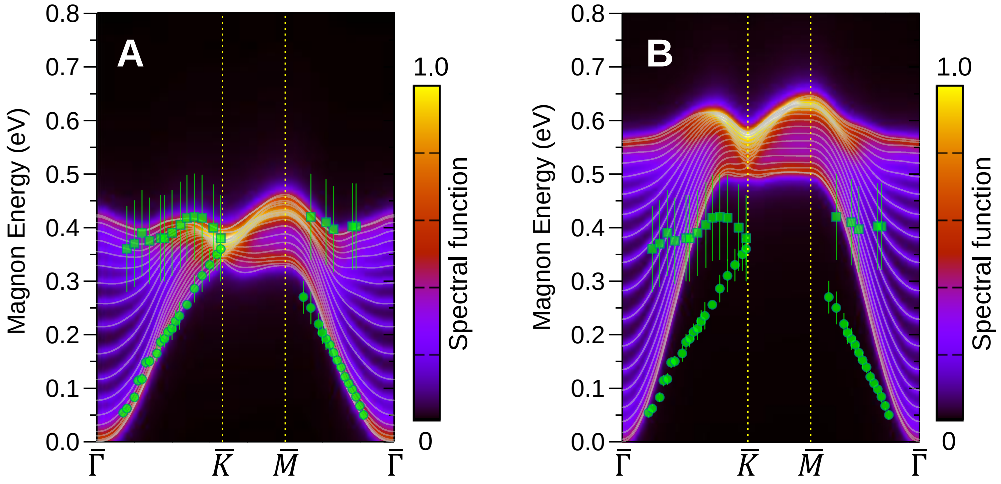

Figure S11. **Magnonic band structure with and without corrections for the effects associated with many-body correlations.** The calculated (solid curves) and measured (symbols) magnon dispersion relation of a 20 ML hcp Co film. (**A**) with and (**B**) without taking the effects associated with the many-body correlations into account. This is accomplished by rigidly shifting the majority electronic bands towards the Fermi level by $\Delta = 0.85$ eV, while keeping the minority bands unaffected. For each case the calculated total MBSF is also shown as the color map.

REFERENCES

1. P. A. M. Dirac, Quantum mechanics of many-electron systems. *Proc. R. Soc. Lond. Ser. A Contain. Pap. Math. Phys. Character* **117**, 610–624 (1928).

2. K. S. Novoselov, A. K. Geim, S. V. Morozov, D. Jiang, Y. Zhang, S. V. Dubonos, I. V. Grigorieva, A. A. Firsov, Electric field effect in atomically thin carbon films. *Science* **306**, 666–669 (2004).

3. A. K. Geim, K. S. Novoselov, The rise of graphene. *Nat. Mater.* **6**, 183–191 (2007).

4. H. Zhang, C. X. Liu, X. L. Qi, X. Dai, Z. Fang, S. C. Zhang, Topological insulators in Bi_2Se_3, Bi_2Te_3 and Sb_2Te_3 with a single Dirac cone on the surface. *Nat. Phys.* **5**, 438–442 (2009).

5. M. Z. Hasan, C. L. Kane, Colloquium: Topological insulators. *Rev. Mod. Phys.* **82**, 3045–3067 (2010).

6. S. M. Young, S. Zaheer, J. C. Y. Teo, C. L. Kane, E. J. Mele, A. M. Rappe, Dirac semimetal in three dimensions. *Phys. Rev. Lett.* **108**, 140405 (2012).

7. A. V. Balatsky, I. Vekhter, J.-X. Zhu, Impurity-induced states in conventional and unconventional superconductors. *Rev. Mod. Phys.* **78**, 373–433 (2006).

8. T. Wehling, A. Black-Schaffer, A. Balatsky, Dirac materials. *Adv. Phys.* **63**, 1–76 (2014).

9. X. Chen, Y. Liu, P. Liu, Y. Yu, J. Ren, J. Li, A. Zhang, Q. Liu, Unconventional magnons in collinear magnets dictated by spin space groups. *Nature* **640**, 349–354 (2025).

10. L. Lu, J. D. Joannopoulos, M. Soljacic, Topological photonics. *Nat. Photonics* **8**, 821–829 (2014).

11. Y. Liu, Y. Xu, W. Duan, Berry phase and topological effects of phonons. *Natl. Sci. Rev.* **5**, 314–316 (2017).

12. Y. Liu, X. Chen, Y. Xu, Topological phononics: From fundamental models to real materials. *Adv. Funct. Mater.* **30**, 1904784 (2019).

13. T. Ozawa, H. M. Price, A. Amo, N. Goldman, M. Hafezi, L. Lu, M. C. Rechtsman, D. Schuster, J. Simon, O. Zilberberg, I. Carusotto, Topological photonics. *Rev. Mod. Phys.* **91**, 015006 (2019).

14. K. Zakeri, Terahertz magnonics: Feasibility of using terahertz magnons for information processing. *Phys. C Supercond. Appl.* **549**, 164–170 (2018).

15. K. Zakeri, Magnonic crystals: Towards terahertz frequencies. *J. Phys. Condens. Matter* **32**, 363001 (2020).

16. H. Yuan, Y. Cao, A. Kamra, R. A. Duine, P. Yan, Quantum magnonics: When magnon spintronics meets quantum information science. *Phys. Rep.* **965**, 1–74 (2022).

17. A. Mook, J. Henk, I. Mertig, Tunable magnon Weyl points in ferromagnetic pyrochlores. *Phys. Rev. Lett.* **117**, 157204 (2016).

18. S. Bao, J. Wang, W. Wang, Z. Cai, S. Li, Z. Ma, D. Wang, K. Ran, Z. Y. Dong, D. L. Abernathy, S. L. Yu, X. Wan, J. X. Li, J. Wen, Discovery of coexisting Dirac and triply degenerate magnons in a three-dimensional antiferromagnet. *Nat. Commun.* **9**, 2591 (2018).

19. W. Yao, C. Li, L. Wang, S. Xue, Y. Dan, K. Iida, K. Kamazawa, K. Li, C. Fang, Y. Li, Topological spin excitations in a three-dimensional antiferromagnet. *Nat. Phys.* **14**, 1011–1015 (2018).

20. Y. Jin, R. Wang, H. Xu, Recipe for Dirac phonon states with a quantized valley Berry phase in two-dimensional hexagonal lattices. *Nano Lett.* **18**, 7755–7760 (2018).

21. L. Chen, J. H. Chung, B. Gao, T. Chen, M. B. Stone, A. I. Kolesnikov, Q. Huang, P. Dai, Topological spin excitations in honeycomb ferromagnet CrI_3. *Phys. Rev. X* **8**, 041028 (2018).

22. B. Yuan, I. Khait, G. J. Shu, F. C. Chou, M. B. Stone, J. P. Clancy, A. Paramekanti, Y. J. Kim, Dirac magnons in a honeycomb lattice quantum *XY* magnet $CoTiO_3$. *Phys. Rev. X* **10**, 011062 (2020).

23. M. Elliot, P. A. McClarty, D. Prabhakaran, R. D. Johnson, H. C. Walker, P. Manuel, R. Coldea, Order-by-disorder from bond-dependent exchange and intensity signature of nodal quasiparticles in a honeycomb cobaltate. *Nat. Commun.* **12**, 3936 (2021).

24. J. A. Schneeloch, Y. Tao, Y. Cheng, L. Daemen, G. Xu, Q. Zhang, D. Louca, Gapless Dirac magnons in $CrCl_3$. *NPJ Quantum Mater.* **7**, 66 (2022).

25. S.-H. Do, K. Kaneko, R. Kajimoto, K. Kamazawa, M. B. Stone, J. Y. Y. Lin, S. Itoh, T. Masuda, G. D. Samolyuk, Damped Dirac magnon in the metallic kagome antiferromagnet FeSn. *Phys. Rev. B* **105**, l180403 (2022).

26. S. Nikitin, B. Fåk, K. W. Krämer, T. Fennell, B. Normand, A. M. Läuchli, C. Rüegg, Thermal evolution of Dirac magnons in the honeycomb ferromagnet $CrBr_3$. *Phys. Rev. Lett.* **129**, 127201 (2022).

27. M. dos Santos Dias, N. Biniskos, F. J. dos Santos, K. Schmalzl, J. Persson, F. Bourdarot, N. Marzari, S. Blügel, T. Brückel, S. Lounis, Topological magnons driven by the Dzyaloshinskii-Moriya interaction in the centrosymmetric ferromagnet Mn_5Ge_3. *Nat. Commun.* **14**, 7321 (2023).

28. M. Terilli, X. Jia, X. Liu, P. Laurell, A. M. Nedić, Y. Chang, T. C. Wu, H. Chen, H. Li, M. H. Upton, J. Kim, J. W. Kim, P. J. Ryan, C. Nelson, J. Zhou, M. Kareev, W. Hu, J. H. Pixley, G. A. Fiete, Y. Cao, J. Chakhalian, Spectrally sharp magnetic excitations above the critical temperature in a frustrated Weyl semimetal. *Nat. Commun.* **16**, 6576 (2025).

29. A. Scheie, P. Laurell, P. A. McClarty, G. E. Granroth, M. B. Stone, R. Moessner, S. E. Nagler, Dirac magnons, nodal lines, and nodal plane in elemental gadolinium. *Phys. Rev. Lett.* **128**, 097201 (2022).

30. A. Scheie, P. Laurell, P. A. McClarty, G. E. Granroth, M. B. Stone, R. Moessner, S. E. Nagler, Spin-exchange Hamiltonian and topological degeneracies in elemental gadolinium. *Phys. Rev. B* **105**, 104402 (2022).

31. G. Sundaram, Q. Niu, Wave-packet dynamics in slowly perturbed crystals: Gradient corrections and Berry-phase effects. *Phys. Rev. B* **59**, 14915–14925 (1999).

32. K. Zakeri, H. Qin, A. Ernst, Unconventional magnonic surface and interface states in layered ferromagnets. *Commun. Phys.* **4**, 18 (2021).

33. P. A. Lindgard, B. N. Harmon, A. J. Freeman, Theoretical magnon dispersion curves for Gd. *Phys. Rev. Lett.* **35**, 383–386 (1975).

34. J. Jensen, A. R. Mackintosh, *Rare Earth Magnetism: Structures and Excitations*, vol. 81 of *International Series of Monographs on Physics* (Clarendon Press, Oxford, UK) (1991).

35. P. Buczek, A. Ernst, L. M. Sandratskii, Different dimensionality trends in the Landau damping of magnons in iron, cobalt, and nickel: Time-dependent density functional study. *Phys. Rev. B* **84**, 174418 (2011).

36. T. Skovhus, T. Olsen, Dynamic transverse magnetic susceptibility in the projector augmented-wave method: Application to Fe, Ni, and Co. *Phys. Rev. B* **103**, 245110 (2021).

37. K. Zakeri, A. Ernst, Dimensionality-driven terahertz Dirac magnons in layered *3d* ferromagnets. *Nano Lett.* **26**, 2660–2665 (2026).

38. S. V. Halilov, H. Eschrig, A. Y. Perlov, P. M. Oppeneer, Adiabatic spin dynamics from spin-density-functional theory: Application to Fe, Co, and Ni. *Phys. Rev. B* **58**, 293–302 (1998).

39. M. Pajda, J. Kudrnovský, I. Turek, V. Drchal, P. Bruno, Ab initio calculations of exchange interactions, spin-wave stiffness constants, and Curie temperatures of Fe, Co, and Ni. *Phys. Rev. B* **64**, 174402 (2001).

40. I. Turek, J. Kudrnovsky, V. Drchal, P. Bruno, Exchange interactions, spin waves, and transition temperatures in itinerant magnets. *Philos. Mag.* **86**, 1713–1752 (2006).

41. H. Okumura, K. Sato, T. Kotani, Spin-wave dispersion of 3*d* ferromagnets based on quasiparticle self-consistent *GW* calculations. *Phys. Rev. B* **100**, 054419 (2019).

42. H. Ebert, S. Mankovsky, S. Wimmer, *Electronic Structure: Metals and Insulators* (Springer International Publishing, 2021), pp. 1–73.

43. R. Vollmer, M. Etzkorn, P. S. A. Kumar, H. Ibach, J. Kirschner, Spin-polarized electron energy loss spectroscopy of high energy, large wave vector spin waves in ultrathin fcc Co films on Cu(001). *Phys. Rev. Lett.* **91**, 147201 (2003).

44. K. Zakeri, J. Kirschner, *Probing Magnons by Spin-Polarized Electrons*, vol. 125 of *Topics in Applied Physics Magnonics from Fundamentals to Applications* (Springer, Berlin, Heidelberg, 2013), chap. 7, pp. 84–99.

45. K. Zakeri, Elementary spin excitations in ultrathin itinerant magnets. *Phys. Rep.* **545**, 47–93 (2014).

46. K. Zakeri, D. Rau, J. Jandke, F. Yang, W. Wulfhekel, C. Berthod, Direct probing of a large spin-orbit coupling in the FeSe superconducting monolayer on STO. *ACS Nano* **17**, 9575–9585 (2023).

47. J. Kirschner, Direct and exchange contributions in inelastic scattering of spin-polarized electrons from iron. *Phys. Rev. Lett.* **55**, 973–976 (1985).

48. K. Zakeri, T. H. Chuang, A. Ernst, L. M. Sandratskii, P. Buczek, H. J. Qin, Y. Zhang, J. Kirschner, Direct probing of the exchange interaction at buried interfaces. *Nat. Nanotechnol.* **8**, 853–858 (2013).

49. K. Zakeri, Y. Zhang, J. Kirschner, Surface magnons probed by spin-polarized electron energy loss spectroscopy. *J. Electron. Spectros. Relat. Phenomena* **189**, 157–163 (2013).

50. E. Michel, H. Ibach, C. M. Schneider, Spin waves in ultrathin hexagonal cobalt films on W(110), Cu(111), and Au(111) surfaces. *Phys. Rev. B* **92**, 024407 (2015).

51. Y.-J. Chen, K. Zakeri, A. Ernst, H. J. Qin, Y. Meng, J. Kirschner, Group velocity engineering of confined ultrafast magnons. *Phys. Rev. Lett.* **119**, 267201 (2017).

52. H. J. Qin, S. Tsurkan, A. Ernst, K. Zakeri, Experimental realization of atomic-scale magnonic crystals. *Phys. Rev. Lett.* **123**, 257202 (2019).

53. K. Zakeri, A. Ernst, Generation and propagation of ultrafast terahertz magnons in atomically architectured nanomagnets. *Nano Lett.* **24**, 9528–9534 (2024).

54. L. Bergqvist, A. Taroni, A. Bergman, C. Etz, O. Eriksson, Atomistic spin dynamics of low-dimensional magnets. *Phys. Rev. B* **87**, 144401 (2013).

55. C. Etz, L. Bergqvist, A. Bergman, A. Taroni, O. Eriksson, Atomistic spin dynamics and surface magnons. *J. Phys. Condens. Matter* **27**, 243202 (2015).

56. A. T. Costa, R. B. Muniz, D. L. Mills, Spin waves and their damping in itinerant ultrathin ferromagnets: Intermediate wave vectors. *Phys. Rev. B* **74**, 214403 (2006).

57. D. L. Mills, *Spin Waves: History and a Summary of Recent Developments*, vol. 1 of *Handbook of Magnetism and Advanced Magnetic Materials* (Wiley & Sons Ltd., 2007), chap. Fundamentals and Theory, pp. 247–282.

58. Y. Zhang, T.-H. Chuang, K. Zakeri, J. Kirschner, Relaxation time of terahertz magnons excited at ferromagnetic surfaces. *Phys. Rev. Lett.* **109**, 087203 (2012).

59. K. Zakeri, A. Hjelt, I. Maznichenko, P. Buczek, A. Ernst, Nonlinear decay of quantum confined magnons in itinerant ferromagnets. *Phys. Rev. Lett.* **126**, 177203 (2021).

60. S. Paischer, D. Eilmsteiner, I. Maznichenko, N. Buczek, K. Zakeri, A. Ernst, P. A. Buczek, Correlations, disorder, and multimagnon processes in terahertz spin dynamics of magnetic nanostructures: A first-principles investigation. *Phys. Rev. B* **109**, l220405 (2024).

61. R. Matsumoto, S. Murakami, Theoretical prediction of a rotating magnon wave packet in ferromagnets. *Phys. Rev. Lett.* **106**, 197202 (2011).

62. K. Zakeri, Y. Zhang, J. Prokop, T. H. Chuang, N. Sakr, W. X. Tang, J. Kirschner, Asymmetric spin-wave dispersion on Fe(110): Direct evidence of the Dzyaloshinskii-Moriya interaction. *Phys. Rev. Lett.* **104**, 137203 (2010).

63. K. Zakeri, Y. Zhang, J. Prokop, T.-H. Chuang, W. X. Tang, J. Kirschner, Magnon excitations in ultrathin Fe layers: The influence of the Dzyaloshinskii-Moriya interaction. *J. Phys. Conf. Ser.* **303**, 012004 (2011).

64. S. Tsurkan, K. Zakeri, Giant Dzyaloshinskii-Moriya interaction in epitaxial Co/Fe bilayers with C_{2v} symmetry. *Phys. Rev. B* **102**, 060406 (2020).

65. K. Zakeri, A. von Faber, Giant spin-orbit induced magnon nonreciprocity in ultrathin ferromagnets. *Phys. Rev. Lett.* **132**, 126702 (2024).

66. K. Zakeri, A. von Faber, S. Mankovsky, H. Ebert, Unraveling the complexity of the Dzyaloshinskii-Moriya interaction in layered magnets: The full magnitude and chirality control. *Adv. Mater.* **37**, e2500152 (2025).

67. H. J. Qin, K. Zakeri, A. Ernst, L. M. Sandratskii, P. Buczek, A. Marmodoro, T. .H. Chuang, Y. Zhang, J. Kirschner, Long-living terahertz magnons in ultrathin metallic ferromagnets. *Nat. Commun.* **6**, 6126 (2015).

68. H. J. Qin, K. Zakeri, A. Ernst, J. Kirschner, Temperature dependence of magnetic excitations: Terahertz magnons above the Curie temperature. *Phys. Rev. Lett.* **118**, 127203 (2017).

69. P. A. McClarty, J. G. Rau, Non-Hermitian topology of spontaneous magnon decay. *Phys. Rev. B* **100**, 100405 (2019).

70. A. B. Schmidt, M. Pickel, M. Donath, P. Buczek, A. Ernst, V. P. Zhukov, P. M. Echenique, L. M. Sandratskii, E. V. Chulkov, M. Weinelt, Ultrafast magnon generation in an Fe film on Cu(100). *Phys. Rev. Lett.* **105**, 197401 (2010).

71. K. Zakeri, J. Wettstein, C. Sürgers, Generation of spin-polarized hot electrons at topological insulators surfaces by scattering from collective charge excitations. *Commun. Phys.* **4**, 225 (2021).

72. T. Balashov, A. F. Takács, W. Wulfhekel, J. Kirschner, Magnon excitation with spin-polarized scanning tunneling microscopy. *Phys. Rev. Lett.* **97**, 187201 (2006).

73. D. Kepaptsoglou, J. Á. Castellanos-Reyes, A. Kerrigan, J. Alves do Nascimento, P. M. Zeiger, K. El Hajraoui, J. C. Idrobo, B. G. Mendis, A. Bergman, V. K. Lazarov, J. Rusz, Q. M. Ramasse, Magnon spectroscopy in the electron microscope. *Nature* **644**, 83–88 (2025).

74. J. Chen, M. Madami, G. Gubbiotti, H. Yu, Magnon confinement in a nanomagnonic waveguide by a magnetic Moiré superlattice. *Appl. Phys. Lett.* **125**, 162403 (2024).

75. K. Zakeri, T. Peixoto, Y. Zhang, J. Prokop, J. Kirschner, On the preparation of clean tungsten single crystals. *Surf. Sci.* **604**, L1–L3 (2010).

76. M. Pratzer, H. J. Elmers, M. Getzlaff, Heteroepitaxial growth of Co on W(110) investigated by scanning tunneling microscopy. *Phys. Rev. B* **67**, 153405 (2003).

77. H. Fritzsche, J. Kohlhepp, U. Gradmann, Epitaxial strain and magnetic anisotropy in ultrathin Co films on W(110). *Phys. Rev. B* **51**, 15933–15941 (1995).

78. M. Etzkorn, P. S. Anil Kumar, W. Tang, Y. Zhang, J. Kirschner, High-wave-vector spin waves in ultrathin Co films on W(110). *Phys. Rev. B* **72**, 184420 (2005).

79. M. Etzkorn, P. S. A. Kumar, J. Kirschner, *High-energy Surface Spin Waves Studied by Spin-polarized Electron Energy Loss Spectroscopy*, vol. 3 of *Handbook of Magnetism and Advanced Magnetic Materials* (Wiley & Sons Ltd., 2007), chap. Spin-polarized electron spectroscopies, pp. 1658–1684.

80. K. Zakeri, C. Berthod, Theory of spin-polarized high-resolution electron energy loss spectroscopy from nonmagnetic surfaces with a large spin-orbit coupling. *Phys. Rev. B* **106**, 235117 (2022).

81. A. I. Liechtenstein, M. I. Katsnelson, V. P. Antropov, V. A. Gubanov, Local spin density functional approach to the theory of exchange interactions in ferromagnetic metals and alloys. *J. Magn. Magn. Mater.* **67**, 65–74 (1987).

82. P. E. Blöchl, O. Jepsen, O. K. Andersen, Improved tetrahedron method for Brillouin-zone integrations. *Phys. Rev. B* **49**, 16223–16233 (1994).

83. L. Szunyogh, B. Újfalussy, P. Weinberger, J. Kollár, Self-consistent localized KKR scheme for surfaces and interfaces. *Phys. Rev. B* **49**, 2721–2729 (1994).

84. K. Zakeri, A. Marmodoro, A. von Faber, S. Mankovsky, H. Ebert, Chirality-inverted Dzyaloshinskii-Moriya interaction. *Phys. Rev. B* **108**, l100403 (2023).

85. F. J. dos Santos, M. dos Santos Dias, S. Lounis, First-principles investigation of spin-wave dispersions in surface-reconstructed Co thin films on W(110). *Phys. Rev. B* **95**, 134408 (2017).

86. A. T. Costa Jr., R. B. Muniz, D. L. Mills, Theory of spin waves in ultrathin ferromagnetic films: The case of Co on Cu(100). *Phys. Rev. B* **69**, 064413 (2004).

87. A. T. Costa, R. B. Muniz, D. L. Mills, Theory of large-wave-vector spin waves in ultrathin ferromagnetic films: Sensitivity to electronic structure. *Phys. Rev. B* **70**, 054406 (2004).

88. S. S. Pershoguba, S. Banerjee, J. C. Lashley, J. Park, H. Ågren, G. Aeppli, A. V. Balatsky, Dirac magnons in honeycomb ferromagnets. *Phys. Rev. X* **8**, 011010 (2018).

89. T.-H. Chuang, K. Zakeri, A. Ernst, L. M. Sandratskii, P. Buczek, Y. Zhang, H. J. Qin, W. Adeagbo, W. Hergert, J. Kirschner, Impact of atomic structure on the magnon dispersion relation: A comparison between Fe(111)/Au/W(110) and Fe(110)/W(110). *Phys. Rev. Lett.* **109**, 207201 (2012).

90. M. A. Ruderman, C. Kittel, Indirect exchange coupling of nuclear magnetic moments by conduction electrons. *Phys. Rev.* **96**, 99–102 (1954).

91. T. Kasuya, A theory of metallic ferro- and antiferromagnetism on Zeners model. *Prog. Theor. Phys.* **16**, 45–57 (1956).

92. K. Yosida, Magnetic properties of Cu-Mn alloys. *Phys. Rev.* **106**, 893–898 (1957).

93. K. Zakeri, J. Prokop, Y. Zhang, J. Kirschner, Magnetic excitations in ultrathin magnetic films: Temperature effects. *Surf. Sci.* **630**, 311–316 (2014).

94. J. Prokop, W. X. Tang, Y. Zhang, I. Tudosa, T. R. F. Peixoto, K. Zakeri, J. Kirschner, Magnons in a ferromagnetic monolayer. *Phys. Rev. Lett.* **102**, 177206 (2009).

95. K. Zakeri, A. von Faber, A. Ernst, Magnons and fundamental magnetic interactions in a ferromagnetic monolayer: The case of the Ni monolayer. *Phys. Rev. B* **109**, l180406 (2024).
